# Mechanism of Bidirectional Leading-Strand Synthesis Establishment at Eukaryotic DNA Replication Origins

**DOI:** 10.1016/j.molcel.2018.10.019

**Published:** 2019-01-17

**Authors:** Valentina Aria, Joseph T.P. Yeeles

**Affiliations:** 1 Division of Protein and Nucleic Acid Chemistry, Medical Research Council Laboratory of Molecular Biology, Francis Crick Avenue, Cambridge CB2 0QH, UK

**Keywords:** DNA replication, replisome, CMG helicase, replication origin, priming, replication fork, leading-strand synthesis, DNA polymerase, primase

## Abstract

DNA replication commences at eukaryotic replication origins following assembly and activation of bidirectional CMG helicases. Once activated, CMG unwinds the parental DNA duplex and DNA polymerase α-primase initiates synthesis on both template strands. By utilizing an origin-dependent replication system using purified yeast proteins, we have mapped start sites for leading-strand replication. Synthesis is mostly initiated outside the origin sequence. Strikingly, rightward leading strands are primed left of the origin and vice versa. We show that each leading strand is established from a lagging-strand primer synthesized by the replisome on the opposite side of the origin. Preventing elongation of primers synthesized left of the origin blocked rightward leading strands, demonstrating that replisomes are interdependent for leading-strand synthesis establishment. The mechanism we reveal negates the need for dedicated leading-strand priming and necessitates a crucial role for the lagging-strand polymerase Pol δ in connecting the nascent leading strand with the advancing replisome.

## Highlights

• Mapping of leading-strand start sites at two *S. cerevisiae* DNA replication origins
• Leading-strand synthesis is established from “lagging-strand” primers
• Pol δ likely plays a key role in establishing all nascent leading strands
• Replisomes remain interdependent until new leading strands are established

## Graphical Abstract

**Figure:**
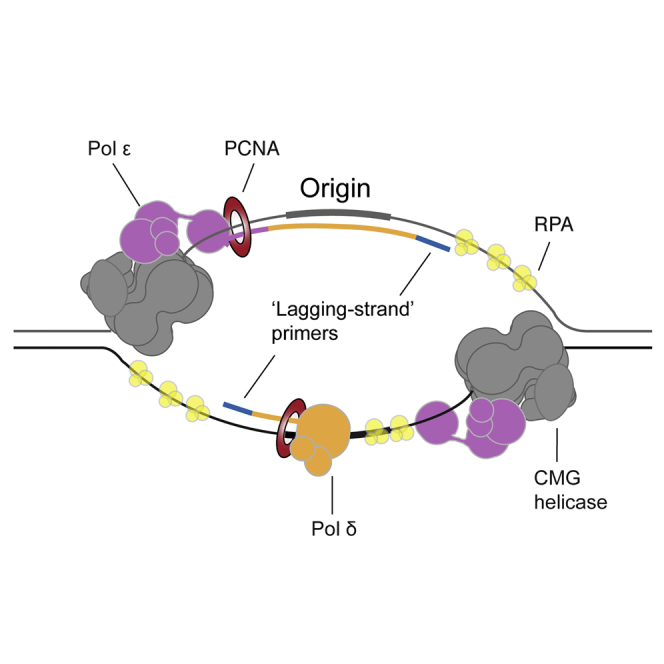

## Teaser

Aria and Yeeles describe the mechanism by which leading-strand replication is established at eukaryotic DNA replication origins. “Lagging-strand” primers, synthesized by replisomes on opposite sides of the origin, are elongated back across the origin by Pol δ until the 3ʹ ends are coupled to Pol ε at the advancing replication forks.

## Introduction

Bidirectional DNA replication is initiated from specific regions of the genome, termed origins. In eukaryotes, assembly of the DNA replication machinery (replisome) begins in the G1 phase of the cell cycle when the ATP-dependent motor component of the replicative helicase, the hexameric Mcm2–7 complex (MCM), is loaded at origins by the origin recognition complex (ORC), Cdc6 and Cdt1 (Bell and Kaguni, 2013, Bell and Labib, 2016). The MCM complex is assembled around double-stranded DNA (dsDNA) as an inactive double hexamer with the N-terminal domains of each hexamer facing one another (Evrin et al., 2009, Remus et al., 2009). Replication commences when double hexamers are activated in S phase to form two Cdc45-MCM-GINS helicases (CMG helicases), around which the replisome is built (Heller et al., 2011, Yeeles et al., 2015). CMG assembly and activation require multiple “firing factors” and are coupled to the initial untwisting and subsequent unwinding of duplex DNA at the replication origin (Douglas et al., 2018). Activated CMG translocates 3ʹ-5ʹ along the leading-strand template in an N-terminus-first orientation (Douglas et al., 2018, Georgescu et al., 2017, Moyer et al., 2006), and consequently, the two CMG complexes must pass one another before extensive template unwinding and DNA synthesis can occur.

Once sufficient single-stranded DNA (ssDNA) has been exposed at origins, synthesis of leading and lagging strands is initiated by the DNA polymerase α-primase complex (Pol α). Lagging-strand synthesis requires repeated cycles of Pol α-dependent priming and subsequent primer extension by Pol δ. Pol α first synthesizes 7–12 nucleotide (nt) RNA primers before transferring them to the DNA polymerase domain, where further extension to about 20–25 nt takes place (Pellegrini, 2012). Evidence suggests that Pol α must be functionally recruited to replication forks for efficient lagging-strand primer synthesis: priming on ssDNA by both human (Collins and Kelly, 1991) and yeast Pol α (Taylor and Yeeles, 2018) is inhibited by RPA; repeated lagging-strand priming by yeast Pol α is dependent on template unwinding by CMG (Georgescu et al., 2015). The details of this functional recruitment are yet to be elucidated. The mechanism by which continuous leading-strand replication is primed by Pol α at replication origins is currently unknown. Furthermore, *in vivo* studies in budding yeast have reached conflicting conclusions regarding the location of leading-strand start sites relative to an origin. For example, one study concluded that the ARS1 origin contains a single leading-strand start site (Bielinsky and Gerbi, 1999). The site was located between the ARS consensus sequence (ACS), which forms part of a high-affinity ORC binding site required for MCM loading (Bell and Labib, 2016, Coster and Diffley, 2017), and the B2 element, a sequence element located downstream of the ACS that enhances origin activity (Chang et al., 2011, Marahrens and Stillman, 1992). However, a second study found that Pol α DNA synthesis peaked just upstream of the ACS, indicating that leading strands might be started outside the origin sequence, potentially from “lagging-strand” primers (Garbacz et al., 2018). Consequently, the relationship between origin sequences and leading-strand start sites is yet to be fully resolved.

Pol ε is responsible for the bulk of leading-strand synthesis *in vivo* (Daigaku et al., 2015, Nick McElhinny et al., 2008, Pursell et al., 2007) and physically associates with CMG (Langston et al., 2014, Sengupta et al., 2013, Sun et al., 2015, Zhou et al., 2017). Furthermore, leading-strand synthesis rates matching those observed *in vivo* can only be attained by a reconstituted replisome when Pol ε is synthesizing the leading strand in conjunction with PCNA (Yeeles et al., 2017). Therefore, once the primer for leading-strand replication has been synthesized, the 3ʹ end must be coupled to CMG-bound Pol ε (CMGE) before rapid and efficient leading-strand replication can commence. Multiple non-mutually exclusive mechanisms might account for this process (Kunkel and Burgers, 2017). The simplest involves direct primer transfer from Pol α to CMGE. Support for this mechanism comes from observations that Pol α can prime the leading-strand template at model replication forks with CMG (Georgescu et al., 2015), and rapid and efficient leading-strand synthesis is observed in *in vitro* replication reactions where Pol α and Pol ε are the only DNA polymerases (Yeeles et al., 2017). In contrast, *in vivo* (Daigaku et al., 2015, Garbacz et al., 2018) and *in vitro* (Yeeles et al., 2017) experiments have indicated that, in addition to its role in lagging-strand synthesis, Pol δ might also participate in the initiation of leading-strand replication via a polymerase switch mechanism, with the 3ʹ end of the nascent leading strand sequentially transferred from Pol α to Pol δ to CMGE. Why such an elaborate mechanism may be required is unknown, as is the frequency by which the two pathways are utilized.

In this study, we have addressed these outstanding questions by mapping start sites for leading-strand replication at two *S. cerevisiae* replication origins using a reconstituted replication system (Taylor and Yeeles, 2018, Yeeles et al., 2015, Yeeles et al., 2017), determining the basis of Pol α recruitment to these sites, and defining the pathway by which the 3ʹ end of the nascent leading strand is connected to CMGE following primer synthesis. This has enabled us to elucidate the mechanism of bidirectional leading-strand synthesis establishment at eukaryotic DNA replication origins.

## Results

### A “Free” Polymerase Couples the Primer for Leading-Strand Replication to CMGE

**Figure 1:**
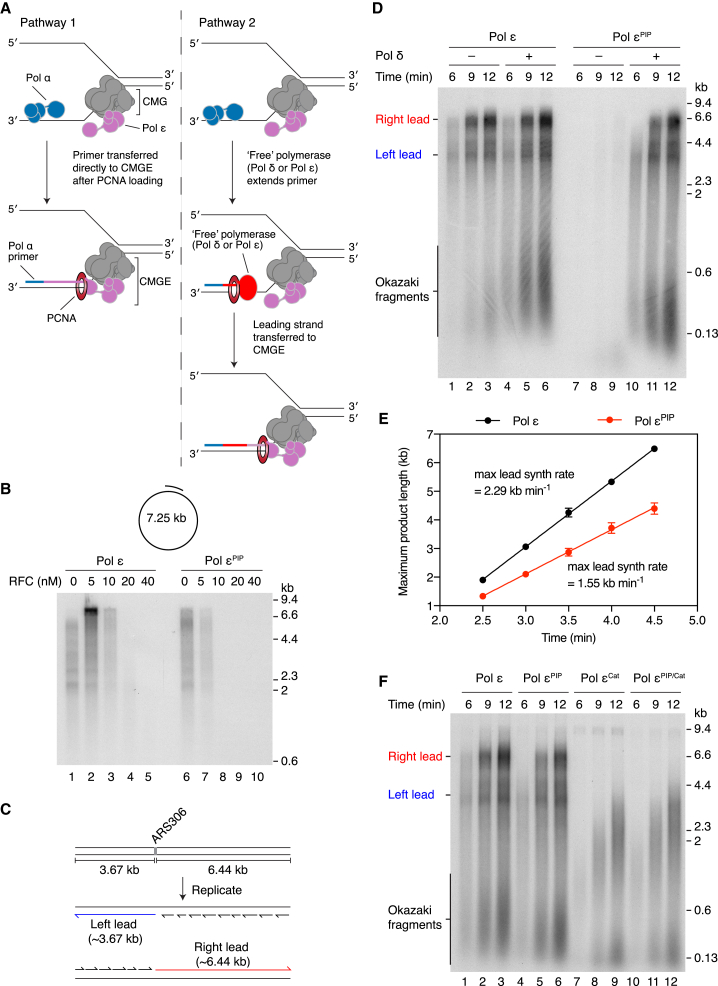
Pol ε^PIP^ Replisomes Are Dependent on Pol δ (A) Diagram illustrating two non-mutually exclusive pathways for connecting the 3ʹ end of the nascent leading strand to CMGE after primer synthesis by Pol α. (B) Primer extension reaction on singularly primed M13 ssDNA. (C) Schematic of the 10.1 kbp ARS306 template used for all replication reactions on naked templates. The putative products of bidirectional origin-dependent replication are illustrated. (D) Standard replication reactions performed on the template illustrated in (C). (E) Quantitation of pulse-chase experiments performed as in Figure S1 in the presence of Pol δ. Error bars represent the standard error of the mean (SEM) from 3 experiments. (F) Standard replication reaction with the indicated Pol ε mutants. Replication products were separated through 0.7% (B) and 1% alkaline agarose gels (D and F).

First, we set out to determine the pathway by which the 3ʹ end of the primer that initiates continuous leading-strand replication is connected to CMGE, considering two non-mutually exclusive pathways (Figure 1A). In pathway 1, the primer is passed directly from Pol α to CMGE via a concerted mechanism. In pathway 2, the primer cannot be transferred directly from Pol α to CMGE; therefore, a “free” polymerase, not associated with CMG, is required to elongate the primer before CMGE takes over. We envisage that Pol δ, rather than Pol ε, would normally fulfill this role by virtue of its greater affinity for primers loaded with PCNA (Georgescu et al., 2014, Schauer and O’Donnell, 2017).

Although previous work showed that Pol δ is dispensable for leading-strand synthesis at replication forks reconstituted from purified *S. cerevisiae* proteins (Yeeles et al., 2015, Yeeles et al., 2017), which might indicate that pathway 1 is operative, omission of Pol δ may not discriminate between the two pathways if free Pol ε is able to substitute in pathway 2, albeit less efficiently. Therefore, to examine the division of labor between pathways 1 and 2 (Figure 1A), we sought to generate a Pol ε mutant compromised for function as a putative free polymerase but not for CMG assembly (Pol ε is an essential firing factor) and replication as part of the CMGE complex. Mutations disrupting the functional interaction between Pol ε and PCNA might fulfill these criteria because PCNA enhances processive primer extension by Pol ε (Chilkova et al., 2007) but is dispensable for both CMG assembly and efficient CMGE-mediated DNA replication (Yeeles et al., 2017). We mutated the Pol ε PCNA interaction motif (PIP box) to generate Pol ε F1199A, F1200A, hereafter referred to as Pol ε^PIP^. Yeast containing this double mutant are reported to grow as wild-type but are sensitive to methyl methanesulfonate (Dua et al., 2002). The primer extension activities of Pol ε and Pol ε^PIP^ were first compared in the presence of RPA, RFC, and PCNA (Figure 1B). Consistent with previous results (Schauer and O’Donnell, 2017), titration of RFC into the reaction stimulated primer extension by Pol ε at a low RFC concentration (5 nM) but inhibited synthesis at higher concentrations, presumably by competing with Pol ε for access to 3′ ends. In contrast, no stimulation of DNA synthesis by Pol ε^PIP^ was observed, and primer extension was inhibited to a greater extent at the higher RFC concentrations (Figure 1B, lanes 6–10). This indicated that Pol ε^PIP^ was compromised for functional interaction with PCNA and, consequently, its ability to function as the putative free polymerase in pathway 2 in the absence of Pol δ (Figure 1A).

We next analyzed Pol ε^PIP^ in an origin-dependent DNA replication reaction (Taylor and Yeeles, 2018, Yeeles et al., 2015, Yeeles et al., 2017). Briefly, MCM double hexamers are loaded onto a 10.1 kbp linear DNA template containing a single *S. cerevisiae* origin of replication (ARS306) (Figure 1C). Subsequent addition of firing factors (S-CDK, Sld3/7, Dpb11, Cdc45, Sld2, Pol ε, GINS, Mcm10) and replication proteins (Pol α, Ctf4, PCNA, RFC, RPA, Pol δ, Mrc1, Csm3/Tof1, Topo I) triggers CMG assembly and activation and the initiation of DNA synthesis. The resulting replisomes perform complete leading- and lagging-strand replication at rates comparable to those measured *in vivo* (Yeeles et al., 2017). Consistent with our previous work (Yeeles et al., 2017), omission of Pol δ from a reaction containing Pol ε only moderately influenced synthesis of the “Left” (∼3.7 kb) and “Right” (∼6.4 kb) leading strands, whereas there was a notable reduction in both the intensity and the length of the Okazaki fragment population (Figures 1C and 1D, compare lanes 1–3 with 4–6). In complete contrast, almost no replication products were observed in the Pol ε^PIP^ reaction in the absence of Pol δ (Figure 1D, lanes 7–9). However, in the presence of Pol δ, Left and Right leading strands and Okazaki fragments were synthesized with efficiency comparable to the Pol ε reaction, confirming that Pol ε^PIP^ is competent for CMG assembly and activation.

At early time points (6 and 9 min), Pol ε^PIP^ produced shorter leading-strand products than Pol ε, indicating that the rate of leading-strand synthesis might be reduced (Figure 1D, compare lane 4 with 10 and 5 with 11). To investigate this in more detail and to determine whether Pol ε^PIP^ contributed to catalysis of leading-strand replication, we performed pulse-chase experiments (Figures 1E and S1A). We previously found that leading-strand synthesis catalyzed exclusively by Pol δ proceeded at approximately one-third the rate of Pol ε-mediated synthesis, and hence replication rates provide information about polymerase usage (Yeeles et al., 2017). In reactions containing Pol ε, leading-strand synthesis proceeded at 2.29 kb min^−1^ (Figure 1E), approximately 20%–40% faster than our previous measurements (Yeeles et al., 2017). The prior experiments were performed on circular templates, and the data therefore indicate that template topology may influence synthesis rates. The rate of leading-strand replication in Pol ε^PIP^ reactions was 1.55 kb min^−^^1^ (Figure 1E), which is considerably faster than the maximum rate supported by Pol δ (0.59 kb min^−1^) (Yeeles et al., 2017). Moreover, the 32% reduction in rate from Pol ε to Pol ε^PIP^ is very similar to the 35% rate reduction that we observed previously when PCNA was omitted from reactions (Yeeles et al., 2017). These findings indicate that catalysis by Pol ε^PIP^ contributes significantly to leading-strand synthesis.

To further validate the participation of Pol ε^PIP^ in leading-strand replication, we created two additional Pol ε mutants: one in which catalysis was disrupted by a point mutation in the polymerase active site (Pol ε^Cat^) (Devbhandari et al., 2017) and a mutant harboring both the PIP box and active site mutations (Pol ε^PIP/Cat^). Replication with Pol ε^PIP/Cat^ proceeded at a similar rate to Pol ε^Cat^ (Figure 1F). In contrast to both Pol ε and Pol ε^PIP^, full-length products of 6.4 kb were not synthesized by 12 min (Figure 1F, lanes 9 and 12), despite replication being initiated within 6 min (Figure 1F, lanes 7 and 10), demonstrating that the rate of synthesis was significantly reduced for these mutants. A pulse-chase experiment with Pol ε^PIP/Cat^ (Figures S1B and S1C) revealed that the elongation rate with this mutant was ∼3-fold slower than Pol ε^PIP^. Collectively, these data show that, despite replication being nearly totally dependent on Pol δ, a polymerase switch occurs at almost all replication forks to enable CMG-bound Pol ε^PIP^ to catalyze the bulk of leading-strand replication. This observation is consistent with data showing that synthesis by Pol ε in the context of the CMGE is significantly more resistant to increasing concentrations of RFC and PCNA than free Pol ε (Schauer and O’Donnell, 2017). Importantly, because Pol ε^PIP^ has a significant defect in primer extension in the presence of RFC and PCNA (Figure 1B), but not leading-strand synthesis in conjunction with CMG (Figures 1D–1F), the data strongly support pathway 2 for replication initiation (Figure 1A), with Pol ε assuming the role of free polymerase in the absence of Pol δ.

### Pol δ Participates in the Establishment of Most Leading Strands

**Figure 2:**
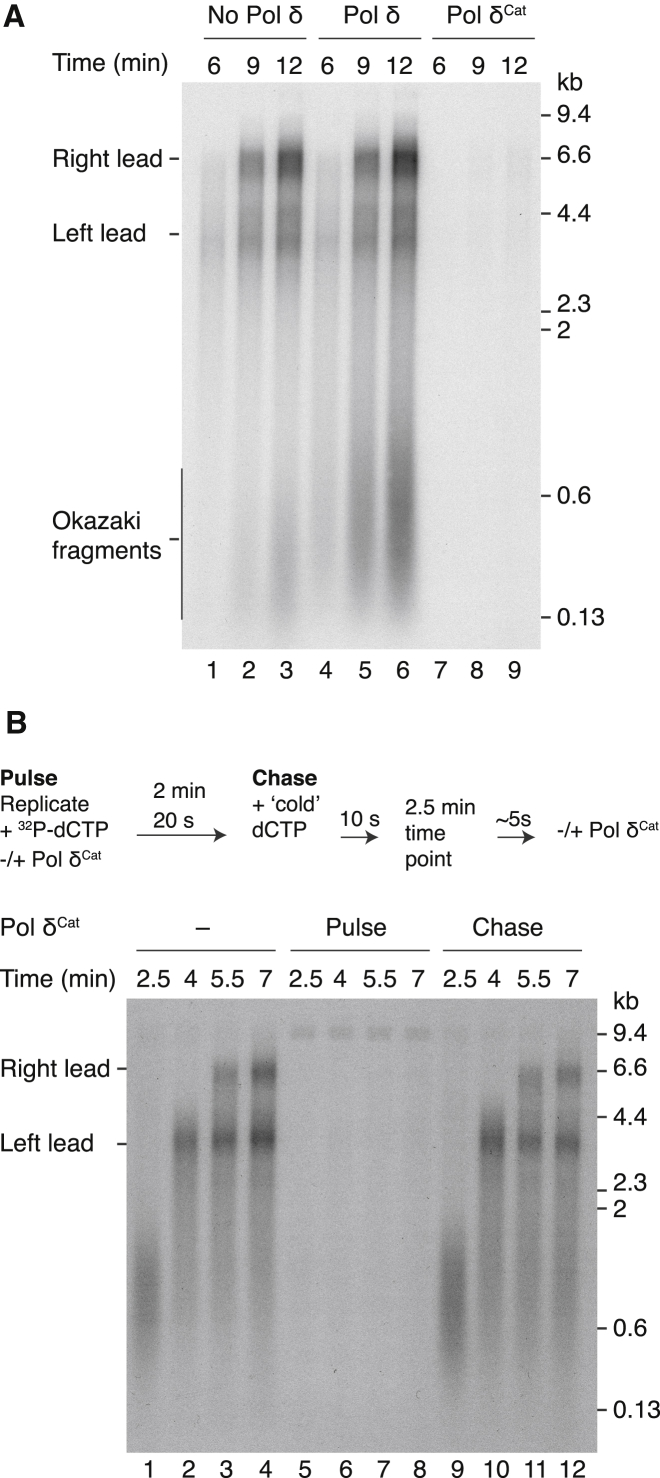
Pol δ Can Access the 3ʹ End of All Nascent Leading Strands (A) Standard replication reaction with the indicated Pol δ proteins. (B) Pulse-chase experiment performed as illustrated. When Pol δ^Cat^ was added during the chase, it was added immediately after the 2.5 min time point. Products were analyzed through 1% (A) and 0.8% (B) alkaline agarose gels.

To assess the frequency of Pol δ participation in leading-strand replication in reactions containing fully-functional Pol ε, we generated a Pol δ catalytic mutant (Pol δ^Cat^). We reasoned that initiation via pathway 1 should be refractory to challenge by this mutant, whereas it should block initiation via pathway 2 (Figure 1A) because Pol δ has a significantly higher affinity than Pol ε for primers loaded with PCNA (Georgescu et al., 2014, Schauer and O’Donnell, 2017). Pol δ^Cat^ almost completely inhibited DNA replication (Figure 2A, lanes 7–9). The extent of inhibition was alleviated by inclusion of Pol δ (Figure S2), indicating that it was a consequence of the mutant protein competing for access to 3ʹ ends. To further examine the mode of inhibition, we performed a pulse-chase experiment in which Pol δ^Cat^ was entirely absent (Figure 2B, lanes 1–4), included in the pulse (lanes 5–8), or added immediately after the chase (lanes 9–12). Figure 2B shows that although Pol δ^Cat^ blocked almost all synthesis when present in the pulse, it had little effect on leading-strand elongation when added after the chase. Thus, once leading-strand synthesis is established, it is largely refractory to challenge by Pol δ^Cat^. We conclude that Pol δ^Cat^ is acting early in reactions to block the establishment of leading-strand replication. Taken together, these results show that Pol δ likely participates in the initiation of most nascent leading strands in our experiments, even in the presence of free Pol ε, and lend further support to pathway 2 being the major pathway for connecting the 3ʹ end of the nascent leading strand with CMGE after primer synthesis at origins.

### Mapping Start Sites for Continuous Leading-Strand Synthesis at ARS306

We next sought to determine the molecular basis underlying the importance of Pol δ in connecting the initiating primer with CMGE. If Pol α is targeted to replication forks to specifically prime the leading-strand template, as has been observed on model replication forks (Georgescu et al., 2015), our data imply that this primer cannot be directly transferred to CMGE. Alternatively, we have recently found that priming by Pol α on the leading-strand template downstream of DNA damage is very inefficient (Taylor and Yeeles, 2018). If this inefficiency is a general—rather than damage-specific—feature of Pol α priming at replication forks, leading-strand synthesis may not be initiated from a leading-strand primer. Rather, synthesis at a bidirectional origin could be established from the first lagging-strand primer made at the replication fork on the opposite side of the origin (Garbacz et al., 2018, Kunkel and Burgers, 2017). This mechanism would presumably require Pol δ to extend the primer back across the origin until it reached the advancing CMGE complex.

**Figure 3:**
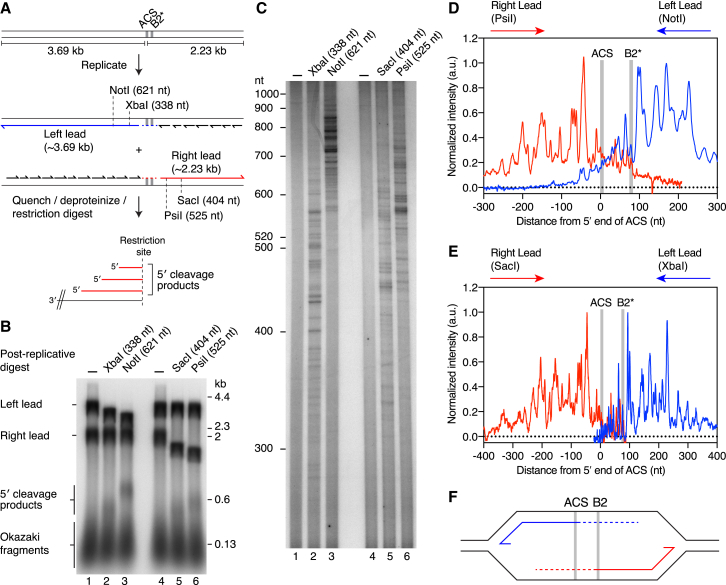
Mapping Leading-Strand Initiation Sites at ARS306 (A) Schematic of the 5.92 kbp ARS306 template used for chromatin replication reactions. Putative replication products are illustrated, and the locations of the restriction enzymes (distances from the 5′ end of the ACS to the cleavage sites) used to truncate the Left and Right leading-strand products are shown. 5′ cleavage products of variable length will be generated if priming occurs at multiple sites (Right lead illustrated). (B) Replication reaction (60 min) on the chromatinized template shown in (A). Products were post-replicatively digested with the indicated enzymes and analyzed through a 1% alkaline agarose gel. The undigested sample was loaded twice (lanes 1 and 4) to serve as a marker. (C) Products from the reaction in (B) were analyzed through a 4% denaturing polyacrylamide gel. (D) Normalized lane profiles for the data in (C) lanes 3 and 6. (E) Normalized lane profiles for the data in (C) lanes 2 and 5. (F) Diagrammatic representation of the data in (D) and (E). Right leading strands (red) are predominantly initiated to the left of the origin and Left leading strands (blue) are mostly initiated to the right. Dotted lines indicate variable start sites for leading-strand replication. The locations of the ACS and a putative B2 element are highlighted with gray bars. The asterisk indicates that the putative B2 element for ARS306 has been assigned based on a match to a B2 consensus sequence (Chang et al., 2011).

To gain insight into the mechanism of priming at eukaryotic replication origins, we devised a strategy to map start sites for continuous leading-strand replication (Figure 3A). Although replication in our system is highly origin specific, as evidenced by distinct leading-strand populations (Figures 1 and 2), the precise location of leading-strand initiation is heterogeneous, which gives rise to diffuse products. We found previously that template chromatinization significantly reduced heterogeneity in leading-strand product length (Taylor and Yeeles, 2018); therefore, we opted to perform mapping experiments on chromatin templates. We first verified that Pol δ was involved in a significant fraction of initiation events on chromatin templates (Figure S3). As with naked templates, addition of Pol δ^Cat^ significantly reduced replication, consistent with Pol δ playing a major role in leading-strand initiation on chromatin. Replication of a 5.92 kbp chromatinized ARS306 template produced two sharp leading-strand products of the predicted sizes (Figures 3A and 3B, lane 1). To map initiation sites, we post-replicatively truncated either the Left or the Right leading strands with enzymes mapping ∼340–620 nt from the ACS (Figures 3A and 3B). Restriction enzyme cleavage liberated short leading-strand fragments that migrated just above Okazaki fragments (Figures 3B, 5′ cleavage fragments, lanes 2, 3, 5, and 6, and S4A and S4B). To better resolve these products, we separated them through a denaturing polyacrylamide gel. Figure 3C shows that distinctive “ladders” were visible in the gel, but not when restriction enzyme was omitted. Importantly, treatment of the reaction products with RNase HII resulted in a downward shift of the ladders of approximately 10 nt (Figure S4C). This shift indicated that the products contained a short section of RNA, as predicted if they represent the 5′ ends of nascent leading strands. Thus, each band in a ladder represents a specific location at which leading-strand replication was started.

To locate start sites relative to the origin, we took lane scans of the data in Figure 3C and normalized the traces relative to the position of the 5′ end of the ACS (see STAR Methods for details). Strikingly, Figure 3D reveals that the Right leading strand was initiated almost exclusively to the left of the ACS, whereas initiation of the Left leading strand occurred predominantly to the right of a putative B2 element (Chang et al., 2011) located ∼70 bp downstream of the ACS. Similar clustering of initiation sites was observed when samples were prepared using alternative restriction enzymes (Figures 3E and S4D and S4E), illustrating that initiation-site assignment was not influenced by the enzyme used for sample preparation. The observed pattern of leading-strand start sites, schematized in Figure 3F, is consistent with leading-strand replication being established from the first lagging-strand primer made at the replication fork on the opposite side of the origin.

### Leading-Strand Start Sites at ARS1

**Figure 4:**
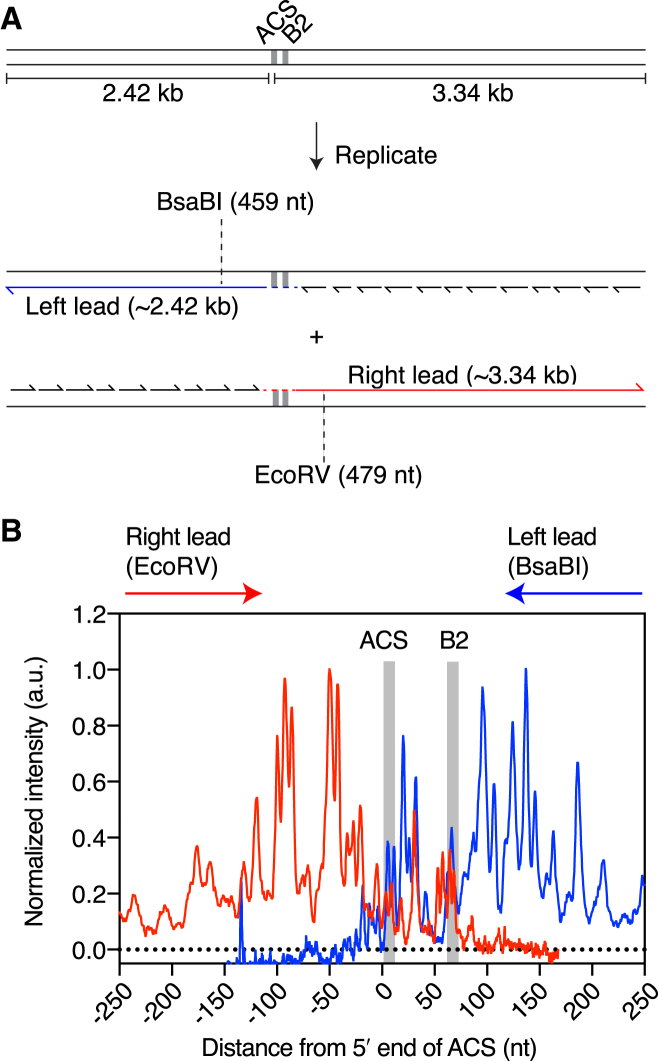
Mapping Leading-Strand Initiation Sites at ARS1 (A) Schematic of the 5.76 kbp ARS1 template used for mapping experiments. Expected replication products are illustrated and the locations of the restriction enzymes used to truncate the Left and Right leading-strand products are shown. (B) Normalized lane profiles for the ARS1 mapping data in Figure S5C in the absence of RNase HII. The locations of the ACS and B2 element are highlighted with gray bars.

We next asked whether the global distribution of initiation sites at ARS306 was specific to this origin or whether it likely represented a general feature of replication initiation at *S. cerevisiae* origins. To this end, we performed equivalent experiments to those in Figure 3 but using a 5.76 kbp template containing the extensively characterized replication origin, ARS1 (Figures 4A and S5). Cleavage of both the Left and Right leading strands again generated ladders of products in the denaturing polyacrylamide gel that were sensitive to RNase HII (Figure S5C). These ladders were highly reproducible between experiments (compare Figures S5C and S5D). As with ARS306, priming sites for the Right leading strand clustered to the left of the ACS, whereas sites for the Left leading strand were mostly distributed to the right of the B2 element (Figure 4). These results indicate that the observed patterns of leading-strand start sites may be a general feature of *S. cerevisiae* origins. Finally, whereas the global pattern of initiation was conserved between the two origins, the precise location of priming sites differed, which may reflect differences in DNA sequence that influence priming-site selection.

### Leading-Strand Replication Is Initiated from “Lagging-Strand” Primers

**Figure 5:**
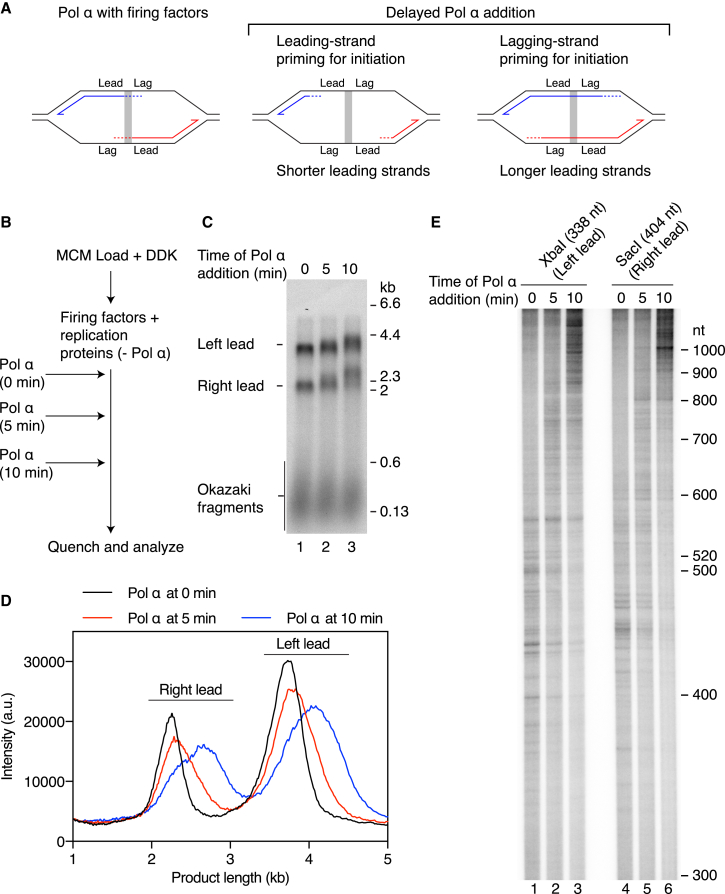
Continuous Leading Strands Are Initiated from Lagging-Strand Primers (A) Experimental design to determine directly whether leading-strand replication is initiated from leading-strand or lagging-strand primers. Delayed addition of Pol α will enable CMG to move away from the origin before synthesis is initiated. If replication is initiated from a leading-strand primer, addition of Pol α after a delay will result in a shortening of leading-strand products. Conversely, if extension of lagging-strand primers from the adjacent replisome is the mechanism used to start leading-strand replication, products will get longer when Pol α addition is delayed. (B) Reaction scheme for delayed addition of Pol α. (C) Replication reaction performed as illustrated in (B) on the chromatinized ARS306 template (Figure 3A) for 60 min. (D) Lane profiles showing the leading-strand replication products in (C). (E) Initiation-site mapping for the experiment in (C).

To test directly whether leading-strand replication is initiated from lagging-strand primers, we devised an experiment in which Pol α was added either together with the firing factors and replication proteins or after a short delay (Figures 5A and 5B). CMG assembly is complete within 5 min under similar experimental conditions (Douglas et al., 2018), and we therefore reasoned that a 5 min incubation in the absence of Pol α would be sufficient to allow CMG assembly and activation and limited helicase movement away from the origin on the majority of templates. In this scenario, initiation of continuous leading strands from lagging-strand primers synthesized on the other side of the origin will result in a lengthening of products, whereas a shortening will be observed if Pol α is recruited specifically to prime the leading-strand template at each fork (Figure 5A). The extent to which product lengths change should be dependent on the time of Pol α addition. Addition of Pol α after a 5 min lag resulted in an appreciable lengthening of both the Right and the Left leading strands compared to when it was added with the firing factors (Figures 5B–5D). This lengthening became even more apparent when Pol α was added after 10 min, and the distribution of leading strands became more diffuse, indicating greater heterogeneity in the position at which they were initiated (Figure 5C, lane 3). Analysis of leading-strand initiation sites confirmed these observations (Figure 5E). For both leading-strand populations, initiation sites closer to the origin (shorter products) were underrepresented when Pol α was added after 5 or 10 min and novel sites further away from the origin were detected (longer products of approximately 600–1,200 nt in length) (Figure 5E). These data directly demonstrate that continuous leading-strand replication is established from lagging-strand primers synthesized by a replisome located on the opposite side of an origin.

### Replisomes Remain Interdependent after CMG Helicase Activation

**Figure 6:**
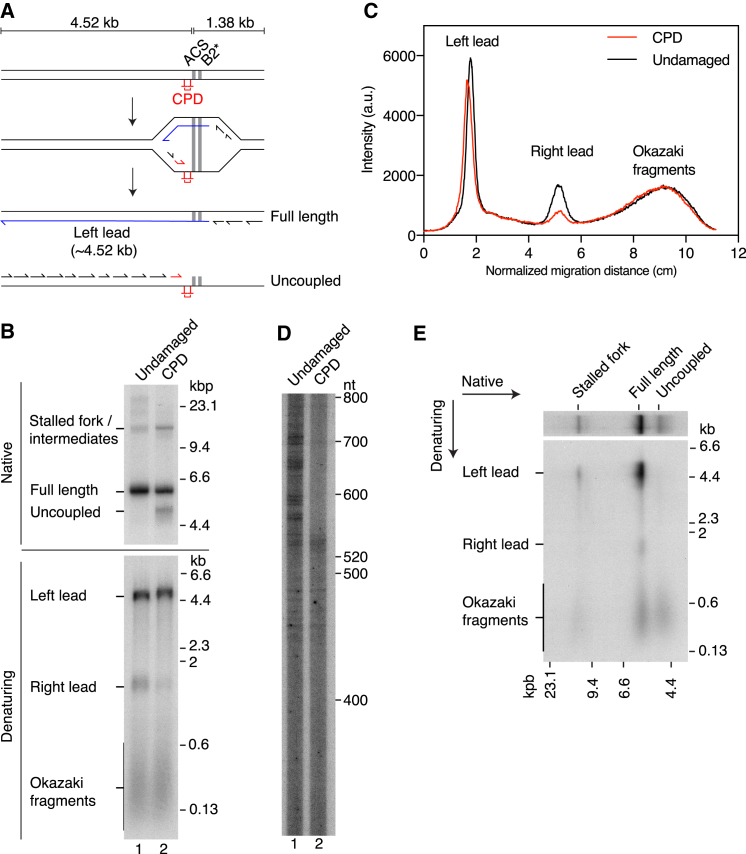
Replisomes Are Interdependent for Leading-Strand Synthesis Establishment (A) Illustration of the ARS306 CPD-containing template and the putative replication products if extension of lagging-strand primers is the sole mechanism to establish leading-strand replication. (B) 60 min replication reaction on undamaged and CPD-containing chromatinized templates. Products were separated through 1% native or denaturing agarose gels as indicated. (C) Lane profiles of the data in (B), denaturing. (D) Products from the reaction in (B) were digested with PsiI and separated through a 4% denaturing polyacrylamide gel. (E) Two-dimensional gel analysis of the products from the CPD-template reaction in (B).

If elongation of lagging-strand primers is the only way to start leading-strand replication, a replisome should be dependent on lagging-strand priming at the adjacent replisome, located across the origin, to start leading-strand synthesis. To investigate this further, we developed an approach to prevent primer elongation back across the origin. We inserted a single cyclobutane pyrimidine dimer (CPD), a potent block to replicative polymerases, 16 nt to the left of the ACS in the lagging-strand template (Figure 6A). The CPD should significantly impact the establishment of Right leading strands if lagging-strand primers are the primary means to establish leading-strand replication. Figure 6B, denaturing, shows that the Right leading strand was significantly underrepresented for the CPD-containing template, and this is further illustrated by the lane profiles for this gel (Figure 6C). Similar results were obtained with a template that produced different size leading-strand products (Figure S6). Analysis of Right leading-strand initiation sites revealed a sharp cutoff for the CPD template, with no products detected above ∼540 nt in length (Figure 6D). The restriction endonuclease used to prepare these samples (PsiI) cuts 543 nt from the CPD. Therefore, the CPD blocks the establishment of leading-strand replication from primers synthesized left of the CPD on the lagging-strand template. Products shorter than ∼540 nt were comparable between the CPD and undamaged templates, demonstrating that priming to the right of the CPD was unaffected by the presence of the lesion. These priming events likely give rise to the residual complete Right leading strands observed for the CPD template (Figures 6B, lane 2, denaturing, 6C, and S6B, denaturing). Importantly, however, there is no evidence for compensatory priming events occurring to the right of the CPD relative to the undamaged template.

Based on our previous work (Taylor and Yeeles, 2018), preventing the establishment of the Right leading strand should cause helicase-polymerase uncoupling, with template unwinding and lagging-strand synthesis continuing to the end of the template to generate “Full-length” and “Uncoupled” products (Figure 6A). Consistent with this idea, products migrating faster than Full-length, which are indicative of Uncoupled products (Taylor and Yeeles, 2018, Yeeles and Marians, 2011), were generated specifically from CPD templates (Figures 6B, native, and S6B, native). As illustrated in Figure 6A, these putative uncoupled products should be composed solely of Okazaki fragments. To examine their composition, we analyzed the products from the CPD template using a two-dimensional gel (Figure 6E). The Full-length product was composed predominantly of Left leading strands and Okazaki fragments with a small amount of Right leading strands that presumably initiated to the right of the CPD (Figure 6D). In contrast, the putative Uncoupled product was composed only of Okazaki fragments. This confirms that although the CPD blocked the establishment of Right leading strands, it did not prevent replication fork progression to the right of the origin. This result demonstrates that in order to establish leading-strand replication, a replisome is dependent on lagging-strand primers synthesized by the replisome on the opposite side of the origin.

## Discussion

In this study, we have determined the pathway by which the 3ʹ end of the nascent leading strand is connected with CMGE after priming, revealing that Pol δ likely plays a crucial role in establishing all continuously synthesized leading strands at eukaryotic replication origins (Figures 1 and 2). Initiation-site mapping experiments have identified start sites for leading-strand replication at two *S. cerevisiae* origins. Synthesis is predominantly initiated outside the origin sequence; Left leading strands are started to the right, and Right leading strands are started to the left (Figures 3 and 4). This distribution strongly suggests that leading strands are established from lagging-strand primers synthesized at replication forks on opposite sides of the origin. We provide direct evidence to support this conclusion: first, delaying Pol α addition to reactions lengthened, rather than shortened, leading-strand products (Figure 5); second, the two replisomes remain interdependent downstream of CMG activation, because placing a single CPD in the lagging-strand template of the leftward fork blocked the establishment of rightward leading strands (Figure 6). The mechanism of priming at origins that we have uncovered provides a clear mechanistic basis for Pol δ function in establishing continuous leading-strand synthesis.

### A Model for Leading-Strand Synthesis Establishment

**Figure 7:**
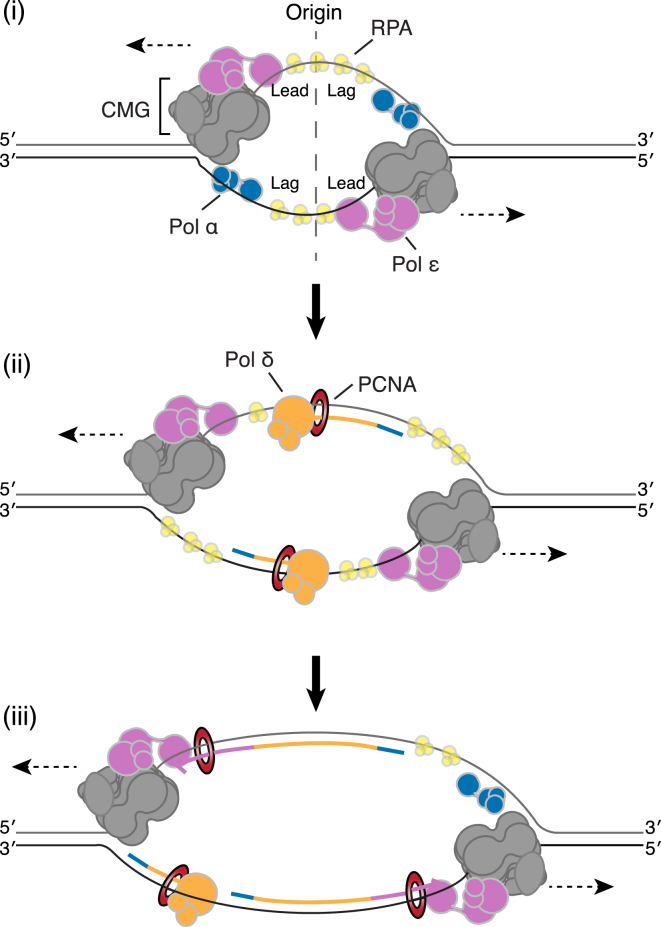
Model Describing the Mechanism of Leading-Strand Initiation at Eukaryotic DNA Replication Origins (i) Following CMG activation, the template is unwound and Pol α is recruited to each replisome to prime on the lagging-strand template. (ii) Pol δ elongates the primers back across the origin until it catches up with the advancing CMG complexes. (iii) A polymerase switch occurs, transferring the 3ʹ end of the nascent strand to CMGE. Dashed horizontal arrows illustrate the direction of CMG movement. The colors of the nascent strands correspond to the colors of polymerases that synthesized them.

Based on the work presented here and current models for CMG helicase activation, we propose the pathway outlined in Figure 7 for the establishment of bidirectional leading-strand replication at eukaryotic DNA replication origins. Following CMG activation, the two helicases translocate past each other (Douglas et al., 2018, Georgescu et al., 2017) and initiate unwinding of a “bubble” of ssDNA at the replication origin. Pol α is functionally recruited to both replisomes, where it primes on the lagging-strand template within a few hundred nucleotides of the origin (Figure 7, i). The two CMGE complexes continue to slowly advance, while at the same time, Pol δ begins elongation of the initial lagging-strand primers (Figure 7, ii). A switch from Pol α to Pol δ also occurs during initiation of leading-strand replication at SV40 origins (Tsurimoto and Stillman, 1991). Because primer extension by Pol δ is significantly faster than the rate of CMG template unwinding (Yeeles et al., 2017), the primers are elongated back across the origin until Pol δ catches up with the advancing CMGE. Although we cannot determine the extent of Pol δ synthesis from our experiments, estimates from polymerase profiling suggest that it is likely to be less than 200 nt *in vivo* (Garbacz et al., 2018). Once Pol δ has connected the 3ʹ end of the leading strand with the replication fork, a polymerase switch occurs to transfer synthesis to CMGE (Yeeles et al., 2017), at which point maximal synthesis rates are established (Figure 7, iii). Further work is required to determine the mechanism of this polymerase switch. It is interesting to note that the switch still occurred efficiently with Pol ε^PIP^, suggesting that a fully-functional interaction between Pol ε and PCNA is not required.

### Priming Leading-Strand Synthesis at Eukaryotic DNA Replication Origins

Our origin-dependent *in vitro* replication system has enabled us to unambiguously map start sites of continuous leading-strand replication. This method has a significant advantage over cellular approaches as it is free from the influences of nucleases, such as FEN1 and Dna2, that process 5ʹ ends of nascent replication products prior to their ligation (Stodola and Burgers, 2017). Furthermore, assignment of start sites for continuous leading-strand replication *in vivo* can be complicated by ligation of Okazaki fragments to the 5ʹ end of the leading strand (Bielinsky and Gerbi, 1999), which does not occur in our system. The patterns of initiation sites that we have determined are consistent with *in vivo* polymerase usage mapping experiments, showing that synthesis by Pol α peaks just upstream of the ACS, with the axis of strand symmetry located 45 nt downstream (Garbacz et al., 2018). However, these experiments could not exclude the possibility that a leading-strand primer was used to initiate synthesis that was subsequently removed by strand-displacement synthesis and 5ʹ flap removal, although the data presented here now suggest that this is unlikely. Zones of leading-strand initiation have also been inferred from Okazaki fragment sequencing data following DNA ligase depletion (McGuffee et al., 2013). The distribution of Okazaki fragments suggested that leading-strand replication was generally initiated somewhere within the nucleosome-free region (NFR), which is broadly consistent with the mechanism presented here. Mapping of initiation sites on an ARS1 containing plasmid in *S. cerevisiae* revealed multiple putative sites clustered such that nascent leading strands overlapped (Bielinsky and Gerbi, 1998), just as our mechanism predicts they must. However, because discontinuous replication products are ligated *in vivo* the authors suggested that the multiple sites likely reflected priming sites of discontinuous lagging-strand synthesis, rather than variable start sites for leading-strand replication (Bielinsky and Gerbi, 1998). In contrast, mapping of start sites at chromosomal ARS1 following prolonged DNA ligase depletion led to the conclusion that ARS1 has a single start site on each strand, located a few nucleotides apart (Bielinsky and Gerbi, 1999). However, these observations are difficult to accommodate with current models of CMG helicase activation (Douglas et al., 2018, Georgescu et al., 2017), and their mechanistic bases remain unclear.

We propose that Pol α is recruited to replication forks to synthesize the initiating primer for continuous leading-strand replication in the same manner it is for regular lagging-strand priming. Importantly, this mechanism obviates the need for a dedicated leading-strand priming mechanism, which may explain why priming on the leading strand downstream of a CPD is very inefficient (Taylor and Yeeles, 2018) and why some eukaryotes encode a dedicated primase-polymerase (PrimPol) for leading-strand re-priming (Bianchi et al., 2013, García-Gómez et al., 2013, Wan et al., 2013). Robust leading-strand priming by Pol α on model replication forks (Georgescu et al., 2015) is not consistent with the data presented here, which may indicate that priming at such model structures is not comparable to nascent-strand priming at origins.

Leading-strand replication is initiated at multiple sites predominantly flanking the origin at both ARS306 and ARS1 in our system. This, together with our discovery that initiation sites can be relocated by delaying addition of Pol α, shows that specific origin sequences are not required for leading-strand initiation. Consistent with this idea, initiation sites are displaced from origins *in vivo* following aberrant transcription termination (Gros et al., 2015). Plasticity in the sequence requirements for leading-strand initiation is likely to be important in higher eukaryotes, where origins show little sequence specificity (Cvetic and Walter, 2005). The use of multiple priming sites is consistent with the relatively relaxed sequence preferences displayed by Pol α (Bullock et al., 1994, Yamaguchi et al., 1985) and is reminiscent of the multiple sites used during the initiation of SV40 and *E. coli* DNA replication (Fang et al., 1999, Hay and DePamphilis, 1982). Initiation sites for a given leading strand were highly reproducible; however, at present, our analysis does not have sufficient resolution to determine the specific nucleotide sequences utilized by Pol α for priming. For both origins, there was a paucity of initiation sites between the ACS and B2, which likely reflects the site of MCM double hexamer loading (Aparicio et al., 1997, Diffley et al., 1994). Initiation sites clustered within regions of approximately 200 nt at either side of the origin. Hence, in this system, priming sites for bidirectional leading-strand replication could be as far as 400 nt apart. However, this may be an overestimate because MCM double hexamers are mobile (Evrin et al., 2009, Gros et al., 2015, Remus et al., 2009), which could introduce heterogeneity in the location of CMG assembly and activation. This may also explain why a small proportion of left and right initiation sites are coincident in our system. The clustering of initiation sites close to the origin indicates that priming occurs rapidly after CMG activation. This is consistent with the high frequency of lagging-strand priming in our reactions, with Okazaki fragments typically being a couple of hundred nucleotides in length. Priming by Pol α on the lagging strand is distributive (Yeeles et al., 2017). If Pol α is recruited to synthesize the first primers via the same mechanism, slow template unwinding prior to the establishment of CMGE-dependent leading-strand synthesis should enhance the probability of priming occurring in the vicinity of the origin. Comparison of our data with nucleosome positioning data for ARS1 (Eaton et al., 2010, Kurat et al., 2017) indicates that priming for leading-strand replication predominantly occurred between the outer boundaries of the −1 and +1 nucleosome positions. Interactions between the replisome and the first nucleosome may also facilitate priming close to the origin as chromatin stimulates regular lagging-strand priming (Kurat et al., 2017).

Elucidating the mechanism of leading-strand synthesis establishment represents an important advancement in our understanding of bidirectional DNA replication initiation at eukaryotic origins. Interestingly, despite significant compositional and structural differences existing between the prokaryotic, viral, and eukaryotic DNA replication machinery (O’Donnell et al., 2013), the use of lagging-strand primers to initiate bidirectional leading-strand replication now appears to be a general mechanism (Bullock et al., 1991, Fang et al., 1999, Hay and DePamphilis, 1982). We speculate that conservation of this process may simply result from the efficient priming required for discontinuous lagging-strand synthesis, negating the need for a dedicated leading-strand priming mechanism to initiate bidirectional DNA replication at origins.

## STAR★Methods

### Key Resources Table

**Table:** 

REAGENT or RESOURCE	SOURCE	IDENTIFIER
**Bacterial and Virus Strains**		

5-alpha Competent *E. coli* (High Efficiency)	New England Biolabs	Cat# C2987H
*Escherichia coli*: Rosetta 2(DE3) strain: F^-^*ompT hsdS*_B_(r_B_^-^ m_B_^-^) *gal dcm* (DE3) pRARE2 (Cam^R^)	Novagen	Cat# 71400

**Chemicals, Peptides, and Recombinant Proteins**		

3X FLAG peptide	Sigma	Cat# F4799
Anti-FLAG M2 affinity gel	Sigma	Cat# A2220
Calmodulin-Sepharose 4B	GE Healthcare	Cat# 17-0529-01
Glutathione Sepharose 4B	GE Healthcare	Cat# 17-0756-01
Talon metal affinity resin	Clontech	Cat# 635502
Ni-NTA Agarose	QIAGEN	Cat# 30210
IgG Sepharose Fast Flow	GE Healthcare	Cat# 17-0969-01
cOmplete, EDTA-free	Roche	Cat# 5056489001
Creatine Phosphate	Sigma	Cat# 27920-1G
Creatine Phosphokinase	Sigma	Cat# C7886-3.5KU
Sephacryl™ S400 High Resolution	GE Healthcare	Cat# 17-0609-10
Microspin G-50 columns	GE Healthcare	Cat# GE27-5330-02
Isw1a	Kurat et al., 2017	N/A
Nap1	Kurat et al., 2017	N/A
Histones	Kurat et al., 2017	N/A
FACT	Kurat et al., 2017	N/A
Nhp6	Ruone et al., 2003	N/A
Cdt1-Mcm2-7	Coster et al., 2014	N/A
ORC	Frigola et al., 2013	N/A
Cdc6	Frigola et al., 2013	N/A
DDK	On et al., 2014	N/A
Sld3/7	Yeeles et al., 2015	N/A
Cdc45	Yeeles et al., 2015	N/A
Dpb11	Yeeles et al., 2015	N/A
Sld2	Yeeles et al., 2015	N/A
GINS	Yeeles et al., 2015	N/A
Pol ε	Yeeles et al., 2015	N/A
S-CDK	Yeeles et al., 2015	N/A
Mcm10	Yeeles et al., 2015	N/A
Pol α	Yeeles et al., 2015	N/A
Ctf4	Yeeles et al., 2015	N/A
RPA	Devbhandari et al., 2017	N/A
Topo I	Yeeles et al., 2017	N/A
Mrc1	Yeeles et al., 2017	N/A
Csm3/Tof1	Yeeles et al., 2017	N/A
RFC	Yeeles et al., 2017	N/A
PCNA	Yeeles et al., 2017	N/A
Pol δ	Yeeles et al., 2017	N/A
Pol ε^PIP^	This study	N/A
Pol ε^Cat^	This study	N/A
Pol ε^PIP/Cat^	This study	N/A
Pol δ^Cat^	This study	N/A

**Experimental Models: Organisms/Strains**		

yCFK1 (Isw1a purification)	Kurat et al., 2017	N/A
yAM33 (Cdt1-Mcm2-7 purification)	Coster et al., 2014	N/A
ySDORC (ORC purification)	Frigola et al., 2013	N/A
ySDK8 (DDK purification)	On et al., 2014	N/A
yTD6 (Sld3/7 purification)	Yeeles et al., 2015	N/A
yJY13 (Cdc45 purification)	Yeeles et al., 2015	N/A
yJY26 (Dpb11 purification)	Yeeles et al., 2015	N/A
yTD8 (Sld2 purification)	Yeeles et al., 2015	N/A
yAJ2 (Pol ε purification)	Yeeles et al., 2015	N/A
yAE37 (S-CDK purification)	Yeeles et al., 2015	N/A
yAE95 (Pol α purification)	Yeeles et al., 2015	N/A
yAE40 (Ctf4 purification)	Yeeles et al., 2015	N/A
yAE42 (Topo I purification)	Yeeles et al., 2017	N/A
yJY32 (Mrc1 purification)	Yeeles et al., 2017	N/A
yAE48 (Csm3/Tof1 purification)	Yeeles et al., 2017	N/A
yAE41 (RFC purification)	Yeeles et al., 2017	N/A
yAE34 (Pol δ purification)	Yeeles et al., 2017	N/A
yVA2	This study	N/A
yVA11 (Pol ε^PIP^ purification)	This study	N/A
yVA7 (Pol ε^Cat^ purification)	This study	N/A
yVA26 (Pol ε^PIP/Cat^ purification)	This study	N/A
yVA28 (Pol δ^Cat^ purification)	This study	N/A

**Oligonucleotides**		

VA_oligo_1: For including C-terminally 3xFLAG to *pol2* gene in yAJ2 strain (Top): AGTATTAC GGTTTTGATATATTATTGAGTTGTATTGCTGA TTTGACCATACGTACGCTGCAGGTCGAC	This study	N/A
VA_oligo_2: For including C-terminally 3xFLAG to *pol2* gene in yAJ2 strain (Bottom) TAAAAAAC GATAGGGTGGCACATCATATTAGGATTAAGAA AATCGATGAATTCGAGCTCG	This study	N/A
VA_oligo_8: For adding D608A mutation in *pol3* gene (Top): GTCCCAATTGCTACTTTGGCTTTCA ACTCTTTGTACCCA	This study	N/A
VA_oligo_9: For adding D608A mutation in *pol3* gene (Bottom): TGGGTACAAAGAGTTGAAAGCC AAAGTAGCAATTGGGAC	This study	N/A
JY_oligo_233: For Pol2 mutation in D640A residue (Top): GTTTGGGTACATAGAAGCAACAGCAACGT GGTAAATCAATGGCA	This study	N/A
JY_oligo_234: For Pol-2 mutation in D640A residue (Bottom): TGCCATTGATTTACCACGTTGCTGTTGC TTCTATGTACCCAAAC	This study	N/A
JY_oligo_212: For Pol-2 mutation in F1199A,F1200A residue (Top): GCTGCTTCTAAGACTAAGAACGTT CCAACTATGG	This study	N/A
JY_oligo_213: For Pol-2 mutation in F1199A,F1200A residue (Bottom): CTTAGTCAAAGAAGTTTGCTTGAAC	This study	N/A
JY_oligo_180: For M13mp18 priming GAATAATGGA AGGGTTAGAACCTACCAT	Yeeles et al., 2017	N/A

**Recombinant DNA**		

pBluescript II KS(-) Phagemid	Agilent Technologies	Cat# 212208-51
M13mp18 Single-stranded DNA	New England Biolabs	Cat# N4040S
ZN3: ARS306 replication template	Taylor and Yeeles, 2018	N/A
pCFK1: ARS1 replication template	Yeeles et al., 2015	N/A
vVA20: ARS306 replication template	This study	N/A
vVA21: ARS306 replication template	This study	N/A
vVA22: ARS306 replication template	This study	N/A
vJY35: pRS304/Pol2 (D640A), Dpb4-Tev-CBP	This study	N/A
vVA1: pRS304/Pol2 (F1199A, F1200A), Dpb4-Tev-CBP	This study	N/A
vVA4: pRS304/Pol2 (D640A, F1199A, F1200A), Dpb4-Tev-CBP	This study	N/A
vVA5: pRS306/Pol31, Pol3 (D608A)	This study	N/A
pCFK1 (Nap1 purification)	Kurat et al., 2017	N/A
pCDFduet.H2A-H2B (Histones purification)	Kingston et al., 2011	N/A
pETduet.H3-H4 (Histones purification)	Kingston et al., 2011	N/A
pRJ1228-Nhp6 (Nhp6 purification)	Ruone et al., 2003	N/A
pAM3 (Cdc6 purification)	Frigola et al., 2013	N/A
vJY19 (PCNA purification)	Yeeles et al., 2017	N/A
pJFDJ5 (GINS purification)	Yeeles et al., 2015	N/A
pTF175 (FACT purification)	Biswas et al., 2005	N/A
pJW22 (FACT purification)	Biswas et al., 2005	N/A
pJM126 (RPA purification)	Addgene	#49339
pET28a-Mcm10 (Mcm10 purification)	Yeeles et al., 2015	N/A
pBP83	Frigola et al., 2013	N/A

**Software and Algorithms**		

ImageJ	National Institute of Health	https://imagej.nih.gov/ij/
Prism 7	GraphPad	https://www.graphpad.com/scientific-software/prism/

### Contact for Reagent and Resource Sharing

Further information and requests for resources and reagents should be directed to and will be fulfilled by the Lead Contact, Joseph Yeeles (jyeeles@mrc-lmb.cam.ac.uk).

### Experimental Model and Subject Details

Proteins were purified from *Saccharomyces cerevisiae* strains (genotype: MATa ade2-1 ura3-1 his3-11,15 trp1-1 leu2-3,112 can1- 100 bar1::Hyg pep4::KanMX) containing integrated expression constructs; or from *Escherichia coli* RosettaTM 2(DE3) cells (Novagen) (genotype: F– ompT hsdSB(rB– mB–) gal dcm (DE3) pRARE2 (CamR)) transformed with plasmids for protein overexpression. Plasmids details are reported in the Key Resources Table.

### Method Details

#### Replication templates

##### Design and construction

The ARS306 templates used in this study were modified from the plasmid ZN3 (Taylor and Yeeles, 2018) using standard molecular biology techniques. The sequence of ZN3 contains approximately 0.2 kbp of genomic DNA right of ARS306. To generate a template more suitable for origin mapping experiments we expanded the genomic DNA sequence on the right of ARS306 to ∼0.8 kbp, and added additional restriction enzyme sites both left and right of the origin to generate plasmid vVA20. A truncated version of this vector, vVA22, was also generated in which approximately 4.2 kbp of DNA was removed. Finally, we generated a template to introduce a single CPD to the lagging-strand template left of ARS306. To do so we modified a derivative of vVA22 that lacked the additional restriction sites to the left of ARS306 to generate vVA21. We inserted a synthetic construct containing two BbvCI restriction sites spaced 9 nucleotides apart, which enables the generation of gapped plasmids for the introduction of a CPD containing oligonucleotide (Taylor and Yeeles, 2018). The BbvCI sites were positioned such that the CPD is located 16 nucleotides to the left of the ACS. The complete sequences of vVA20, vVA21 and vVA22 are listed below.

To generate linear replication templates all plasmids were purified with CsCl gradients, linearized at unique restriction sites and processed as previously indicated (Taylor and Yeeles, 2018). Experiments were performed on the following templates: Figures 1, 2, S1 and S2 were performed on NruI linearized vVA20; Figures 3, 5, S3 and S4 were performed on PvuI linearized vVA22; Figures 4 and S5 were conducted on AlwNI linearized pCFK1 (Yeeles et al., 2015); Figures 6 and S6 were performed with undamaged and CPD versions of vVA21 linearized with AlwNI and PvuI respectively.

##### vVA20 sequence

CTGACGCGCCCTGTAGCGGCGCATTAAGCGCGGCGGGTGTGGTGGTTACGCGCAGCGTGACCGCTACACTTGCCAGCGCCCTAGCGCCCGCTCCTTTCGCTTTCTTCCCTTCCTTTCTCGCCACGTTCGCCGTCCTTCAATGAAACATCGTTGGCCACTAATTTGGCCAGTGCAAAGTAGAACAAATCGGCAGCCTCCCAAGAAAGCTCCTTCTTACCCTTTGCCTCAGTCAGTTCTTCAGCTTCTTCCTTGATCTTGGCATCTAACAATGCAGAGTCGTTGAATAGTCTTCTAGTATAAGATTCCTCTGGAGCGTCCTGTAGCCTTTGTTTTAGTAAAGATTCTAGCCCCACCAAACCATGCTTGAATTCACCAAAGCAAGACATGGTCTCCAAGTGGCAAAATCCAACGTTTTCTTGTTCAACGATAAACTTTAAGGCATCCGAATCACAGTCAGTAGAGATTTGTAAAAGCTTTTGGCCATTGCCAGAAGTTTCACCCTTGATCCAGATTTCATTCCTAGAACGAGAATAATAAACGCCACGACCCAATTCGATGGCCTTTGCTATAGATTTCTTCGAAGAATACACCAACCCTAGACAACGCTCATATTGGTCCACAACTAGGGTGGTATATAAACCGTCAGGACGGTCTGTACGTACTTCACCAAGCACTTCTTTGGTCAACATATCCTTGCTTAATTTCTTTATGGACACAATTTTATCTTGCGAGAATTTTTGTTTTACCATGAATTGATTGGAGAAAACACCGTTCTCTTCCACAACAACACGCTCCTTTGGTACATTCAATTGTTCAACCAAGTGTTCGGCTGTTTTAGCATCTTGGCTTGCAATGAACAGAGAAGAAACTCCGTTGTTCAAGAAGGCAATGATTTCATCATCGCTGAATTTACCACTTGGCAAGGACAAAGCCACCAATGGAACTTCTTCCTCTTTGGAGAACTGGAGAATCTCTTCATTACTCAGGCTCGAGCCATCCAAAAGTACCTGACCAACAAGTGAAACGTATTCCTTCTTACTATTCCATGAGGCCAGATCATCAATTAACGGTAGAATCGGCAAAACCATTATTCAGAAAAAAAATTTTGTAAACTATTGTATTACTATTACACAGCGCAGTTGTGCTATGATATTAAAATGTATCCAGAACACACATCGGAGGTGAATATAACGTTCCATATCTATTATATACACAGTATACTACTGTTCATAGTCATATCCTTTTCTTACCTTCTATATCGAATGACTGATAATGCAACGTGAGTCACTGTGCATGGGTTTAGCAATTATTAAACTAATTTACCGGAGTCACTATTAGAGTCAGTTCGACTGCCTAGAAGAACTGCTGGTTGTCAGGATTGTGATGGGGGCATTCTGCTGTATTATGACCCATCGTATCGCAATGCTCACACCACTGTTGTCTTCCTGCCGTGGTATCGACTGGTGCAGGGGGGTCGAAAATTGGCAACGATTCCACGGCTGTTTGTGCTTGAGCCTGTTCCAACTGTTTGAACCTTTCATTAGCCTCTTCAAGTTTTTTCGTTAAGGATGCCACCTCTTCCGATGAGGAATCTTGTGGTTTTGTCAAAAATAGTTCCTTGCTCAAATTTTGGTATTCTTTACTGAGCGAATCGTTATGCATTTTCAATTGTTCGCGTTCTTTAGCCCACTTTGTCTTGTGTAACTCAAATTGGTCTTCTATGTTGCGTAATTGTTCCAGCTGTTTTTTCAGGAGTTCGACATCTTCGTTGGCACCAGTGGGTTGATTATGAGAAAGATTTCTCTCTTCGTTTTCTTTGATCTCTTCGTGTAGTTGGCTTACGACAGCAAGTAGCTGTTCATTCTCAGCGTCAAAAAACTGCTTTTGTTTGGCTTGCTGTCTGCGTTCGAGCAGACATTGTTGCTTGAGATGGTCTATCTCTTTCTCTCTTTCTTGTATTGTGGCTTCATACCTATCAAAAGTCGGTTGCACTTCTTCGAGGACCATTCTTTGGTCATCGAGTAGCCTTTTGTAGTGTAGTTGTTTCCTTTGTAGCTTTTCGATGGTCAATTGGCGATCGCGTAATTCAATTGTAACTTCGCTGCTATTGAGGTCATTCATGTGGCCATTGTCCGGTTTCCAATCGCTGGTGGTGTTGTGATTAGCCTTTCTGTCTGATGACAGGATAGAGTCCACCTCCATTCTGTCTTCTCTGTTATCGTAACCAAATTCTTGCTGTTGATGGTGATCCGATGCCTCCTGGTCCATCGACTGTTGATTACCGCTGTGCCGACTGGTGATCCGGAAACTTCTCATGGGTGTGGGGGATTTAGGATCATCCATGGGAGAGAAGCGCTTAGTGAGCCTCACAATAGATCTGTTCACGGGTATTGATAGCGGTTCCATTGTCGTTCTTCTCGAGGTTTGCCATATCGGTCCGTTCTCGATCAATGATGCGACTTTTTGCAACTGAATAAATAGTCCACTTTGAGGATACTCCGTTTGAAAATACTTCTTCCCCTAGGAATGATCCATCGTTCTTACCAATGTTGGCAAGTAAGTCTACACCAGCAAACATTCCACGCGTCGTGTCCACTGGACCCACGTATTTCAGTTGTCCGCGGCCGAAATTTGGGATTTGGTTTAAACATCCTATCTTTCTTTGATATCTATCCATGGTATATTAAGCGCATACGGCGCCAGCCACTAGTCAACGCCTTTTACCTTGTCCTTTGATGCATGCCTCGTCCAAACGTTTTTGGTGTCTTGGCCAATTGCCCTTCTGAAAAATCTCACTGTCCGCAACTCATTAAAAGATACCCAAGCAAGCTACACGATAAAGAAAGGAGAAAGTTCATTACTGGAACGTACATATAGCGATACAAACGTATAGCAAAGATCTGAAATGGATACGGATAAGTTAATCTCAGAGGCTGAGTCTCATTTTTCTCAAGGAAACCATGCAGAAGCTGTTGCGAAGTTGACATCCGCAGCTCAGTCGAACCCCAATGACGAGCAAATGTCAACTATTGAATCATTAATTCAAAAAATCGCAGGATACGTCATGGACAACCGTAGTGGTGGTAGTGACGCCTCGCAAGATCGTGCTGCTGGTGGTGGTTCATCTTTTATGAACACTTTAATGGCAGACTCTAAGGGTTCTTCCCAAACGCAACTAGGAAAACTAGCTTTGTTAGCCACAGTGATGACACACTCATCAAATAAAGGTTCTTCTAACAGAGGGTTTGACGTAGGGACTGTCATGTCAATGCTAAGTGGTTCTGGCGGCGGGAGCCAAAGTATGGGTGCTTCCGGCCTGGCTGCCTTGGCTTCTCAATTCTTTAAGTCAGGTAACAATTCCCAAGGTCAGGGACAAGGTCAAGGTCAAGGTCAAGGTCAAGGACAAGGTCAAGGTCAAGGTTCTTTTACTGCTTTGGCGTCTTTGGCTTCATCTTTCATGAATTCCAACAACAATAATCAGCAAGGTCAAAATCAAAGCTCCGGTGGTTCCTCCTTTGGAGCACTGGCTTCTATGGCAAGCTCTTTTATGCATTCCAATAATAATCAGAACTCCAACAATAGTCAACAGGGCTATAACCAATCCTATCAAAACGGTAACCAAAATAGTCAAGGTTACAATAATCAACAGTACCAAGGTCGCGACGGTGGTTACCAACAACAACAGGGACAATCTGGTGGTGCTTTTTCCTCATTGGCCTCCATGGCTCAATCTTACTTAGGTGGTGGACAAACTCAATCCAACCAACAGCAATACAATCAACAAGGCCAAAACAACCAGCAGCAATACCAGCAACAAGGCCAAAACTATCAGCATCAACAACAGGGTCAGCAGCAGCAACAAGGCCACTCCAGTTCATTCTCAGCTTTGGCTTCCATGGCAAGTTCCTACCTGGGCAATAACTCCAATTCAAATTCGAGTTATGTGTACACGCAACAGGCTAATGAGTATGGTAGACCGCAACAGAATGGTCAACAGCAATCCAATGAGTACGGAAGACCGCAATACGGCGGAAACCAGAACTCCTAAGGACAGCACGAATCCTTCAATTTTTCTGGCAACTTTTCTCAACAGAACAATAACGGCGCGCCGAACCGCTACTGAACGATGATTCAGTTCGCCTTCTATCCTAAGTTTACGTATTTGCTAGCGCATATAACTTAGCGGGAAATTATTAATTGACCGGTAGGACAATTTTGTTGCACGTGATGCCTCAATCGTCTGCTTGCTTCCATAGTTAACATGAGGATCCGCAGTACCAACCTCAGCACTTAAGTCCTCAGCGCAGTACCAACTGCAGGATGCCCTTTTTGACGTATTGAATGGCATAATTGCACTGTCACTTTTCGCGCTGTCTCATTTTGGTGCGATGATGAAACTTTCATGAAACGTCTGTAATTTGAAACAAATAACGTAATTCTCGGGATTGGTTTTATTTAAATGACAATGTAAGAGTGGCTTTGTAAGGTATGTGTTGCTCTTAAAATATTTGGATACGACATCCAAAATCTTTTTTCCTTTAAGAGCAGGATATAAGTCGACAAGTTTCTGAAAATCAAAATGGTAGCAACAATAATGCAGACGACAACAACTGTGCTGACGACAGTCGCCGCAATGTCTACTACCTTAGCATCCCATTACATATCTTCGCAAGCTAGTTCCTCGACGAGTGTAACAACAGTAACGACAATAGCGACATCAATACGCTCTACACCGTCTAATCTACTCTTTTCTAATGTGGCGGCTCAGCCAAAATCATCTTCAGCAAGCACAATTGGGCTTTCAATCGGACTTCCCATCGGAATATTCTGTTTCGGATTACTTATCCTTTTGTGTTATTTCTACCTTAAAAGGAATTCGGTGTCCATTTCAAATCCACCCATGTCAGCTACGATTCCAAGGGAAGAGGAATATTGTCGCCGCACTAATTGGTTCTCACGGTTATTTTGGCAGAGTAAGTGTGAGGATCAGAATTCATATTCTAATCGTGATATTGAGAAGTATAACGACACCCAGTGGACCTCGGGTGATAACAAGTCTTCAAAAATACAGTACAAAATTTCCAAACCCATAATACCGCAGCATATACTGACACCTAAGAAAACGGTGAAGAACCCATATGCTTGGTCTGGTAAAAACATTTCGTTAGACCCCAAAGTGAACGAAATGGAGGAAGAGAAAGTTGTGGATGCATTCCTGTATACTAAACCACCGAATATTGTCCATATTGAATCCAGCATCCCCTCGTATAATGATTTACCTTCTCAAAAAACGGTGTCCTCAAAGAAAACTGCGTTAAAAACGAGTGAGAAATGGAGTTACGAATCTCCACTATCTCGATGGTTCTTGAGGGGTTCTACATACTTTAAGGATTATGGCTTATCAAAGACCTCTTTAAAGACCCCAACTGGGGCTCCACAACTGAAGCAAATGAAAATGCTCTCCCGGATAAGTAAGGGTTACTTCAATGAGTCAGATATAATGCCTGACGAACGATCGCCCATCTTGGAGAGCATACGACTACCGCCGTATAATAACACGCCTCTGGATGCAAATGACAGTGTGAATAACTTGGGTAATACCACGCCAGATTCACAAATCACATCTTATCGCAACAATAACATCGATCTAATCACGGCAAGACCCCATTCAGTGATATACGGTACTACTGCACAACAAACTTTGGAAACCAACTTCAATGATCATCATGACTGCAATAAAAGCACTGAGAAACACGAGTTGATAATACCCACCCCATCAAAACCACTAAAGAAAAGGATATAAAGAAGACAAAGTAAAATGTATCAGCATTTACAACATTTGTCACGTTCTAAACCATTGCCGCTTACTCCAAACTCCAAATATAATGGGGAGGCTTGCGTCCAATTAGGGAAGACATATACAGTTATTCAGGATTACGAGCCTAGATTGACAGACGAAATAAGAATCTCGCTGGGTGAAAAAGTTAAAATTCTGGCCACTCATACCGATGGATGGTGTCTGGTAGAAAAGTGTAATACACAAAAGGGTTCTATTCACGTCAGTGTTGACGATAAAAGATACCTCAATGAAGATAGAGGCATTGTGCCTGGTGACTGTCTCCAAGAATACGACTGATGAAAATAATATTGACGTTCGCATTTAATCTATACCTATAATTCTGTACTTATATACTGTTCCTTAATTGAAGATTTCAACATCGTTTTTGATGTAGGTCTTTTCACCTGGAGGTGCGGCTGGGCTACCGAAGACTAATTGAGCTTGTACGGTCCAAGACTCAGGGATTTTGCTTGGCAAAGCAGCTTTTATGTAACCATTGTAGTGTTGTAGGTGACCACCCAGGCCCATTGCCTCCAAGGCAACCCACGAGTTGATTTGAGCGGCACCAGAGGTATGGTCCGCGAAACTAGGGAATGCAGCTGCGTACGCTGGGAAGTCAGCCTTTAGCTTTTCAGTTACCTTGTCGTCGGTGAAGAAGATTACAGAACCAAAGGCCTCATCCCTTGCTGAAGCAGGCCTCTTTTGACCGGCAGGGCTTTCTATAGCCTTAGTCACTTCGTCCCAAACTTTTTTGTGAGTTTCACCAGTCAAGATAACAGCGCGATTTGGCTGGGAGTTGAAAGCGGTGGGTGTGGCTCGAATGATGGTTTGGACGACGGATTGGATGTCGTTGATAGTAATTTCACCAGGTGCGGCCGCTTTCAAAGCGTAAATAGTACGACGAGCAGTTAAAGTTTTCAAATAAGTTGCAACAGCAGACATGATATTGGATTGCCGGAATGGCGATATGTTGATCCCGGATACTTCAGTCTACGAAAAAAGTACAAATTATGTGTCAGTTCCTTCAGTATGGTGTCCTTATATACTGTAGTTTGGACAAGGTGCAAATGCCAAGACCCTAGCCCGAAAAGCTCGAGGCACCCCAGGATCTTCCCCTTTACGTAATTTTCACGTAAAACGCCACAGTCCGATTTTTCTAGAATAATCATTAGTAAAAGCGGTATACTGGATTATTGTACGATAACAAGGTAGAGCTTTATTACTAAGCTAAGACGTTCTTACATCAATAGTGCTGTTCGTTATTGACGTCAGGAGAAGGAGCGGGTCTGGTGAATAGTGTAAGCAGTGTTTCTGAACTTTTTCTTCGTCTAAGTCCTTGTAATGTAAGGTAAGAATGCAAGCATCTTGTTTGTAACCCGGGTGTACGTTGACGTTAGTAAGGGGTGTACGTTGACGTTAGTAAGTCACAAACCCAAGCTTAACTTCTTCGTGAGGAAGGAAAGTGTTGTCTCCTACTTTTTTCAAATTTTCGAATTGTATTTATATTTATTTAGTACTTCTTGAGTTTACATATCCTTCGTAAAAATGCAACTTTTGTCGAAAAACACTTCCAAAAAAAAATAATAATGAATTTATGAAGCATACTAACGAGCGAGCACATCGCTGACCTATCATTACTTCATGAGATAAATTAAGATCTCCTCATATGCGAATTTCCTGTTCAGTGATAAACGTTGATTACGTTATTGATAAAAGTCTTTTCTTCTGGCAAGGGGTACCTGGAACACCAAAGACCAATTGAGATTGTACAGTCCACGCAATAGGAACATCTTGAGGCAAAGCAGATTTGACGTAGTCATTATAGTGTTGCAAATTAGCCCCCAATCCCAATAGTTCGAGGGCAGTCCAAGACTGAATTTGCACAGCACCGGTCGTATGAGCTCGCATGTTGGGAAAGCGGCTGCCAAGGCTGGAAATCTCTTTGCAGTTTTTCAGTTGGTCCTTCATCAGTGAAGAAAATGACTGAACCGTAAGCCTCATCTCTGCAAGACTCTGGTCTCTTATAAAGCAGTTGGCATTGCGCTCGCAACAGCATCCCATATCCTTTTGTGTGTATCACCAACGATAATGACAGCGCGATTCACTTGTGAGTTAAAAGCTGTTGGCGTATTCTTGAGAATAACGTGTACAGTTCTCTTTACATCATCCAAACCGACACCTTGTGGTAATTCGGGCTTCAAATTGTAGATGGTACGACGGTTTGTAATAGCGTTTAAGTAGTTTCCAGTTGGGGACATTTCTTTGGCTTGGAGGTCTGGTGTTCTTGATTTTGATGGTGTATATAGCTTTAAAAAACCAAAAATGATCAACCTTTATATCGCTCTTCCGCTTCCTCGCTCACTGACTCGCTGCGCTCGGTCGTTCGGCTGCGGCGAGCGGTATCAGCTCACTCAAAGGCGGTAATACGGTTATCCACAGAATCAGGGGATAACGCAGGAAAGAACATGTGAGCAAAAGGCCAGCAAAAGGCCAGGAACCGTAAAAAGGCCGCGTTGCTGGCGTTTTTCCATAGGCTCCGCCCCCCTGACGAGCATCACAAAAATCGACGCTCAAGTCAGAGGTGGCGAAACCCGACAGGACTATAAAGATACCAGGCGTTTCCCCCTGGAAGCTCCCTCGTGCGCTCTCCTGTTCCGACCCTGCCGCTTACCGGATACCTGTCCGCCTTTCTCCCTTCGGGAAGCGTGGCGCTTTCTCATAGCTCACGCTGTAGGTATCTCAGTTCGGTGTAGGTCGTTCGCTCCAAGCTGGGCTGTGTGCACGAACCCCCCGTTCAGCCCGACCGCTGCGCCTTATCCGGTAACTATCGTCTTGAGTCCAACCCGGTAAGACACGACTTATCGCCACTGGCAGCAGCCACTGGTAACAGGATTAGCAGAGCGAGGTATGTAGGCGGTGCTACAGAGTTCTTGAAGTGGTGGCCTAACTACGGCTACACTAGAAGGACAGTATTTGGTATCTGCGCTCTGCTGAAGCCAGTTACCTTCGGAAAAAGAGTTGGTAGCTCTTGATCCGGCAAACAAACCACCGCTGGTAGCGGTGGTTTTTTTGTTTGCAAGCAGCAGATTACGCGCAGAAAAAAAGGATCTCAAGAAGATCCTTTGATCTTTTCTACGGGGTCTGACGCTCAGTGGAACGAAAACTCACGTTAAGGGATTTTGGTCATGAGATTATCAAAAAGGATCTTCACCTAGATCCTTTTAAATTAAAAATGAAGTTTTAAATCAATCTAAAGTATATATGAGTAAACTTGGTCTGACAGTTACCAATGCTTAATCAGTGAGGCACCTATCTCAGCGATCTGTCTATTTCGTTCATCCATAGTTGCCTGACTCCCCGTCGTGTAGATAACTACGATACGGGAGGGCTTACCATCTGGCCCCAGTGCTGCAATGATACCGCGAGACCCACGCTCACCGGCTCCAGATTTATCAGCAATAAACCAGCCAGCCGGAAGGGCCGAGCGCAGAAGTGGTCCTGCAACTTTATCCGCCTCCATCCAGTCTATTAATTGTTGCCGGGAAGCTAGAGTAAGTAGTTCGCCAGTTAATAGTTTGCGCAACGTTGTTGCCATTGCTACAGGCATCGTGGTGTCACGCTCGTCGTTTGGTATGGCTTCATTCAGCTCCGGTTCCCAACGATCAAGGCGAGTTACATGATCCCCCATGTTGTGCAAAAAAGCGGTTAGCTCCTTCGGTCCTCCGATCGTTGTCAGAAGTAAGTTGGCCGCAGTGTTATCACTCATGGTTATGGCAGCACTGCATAATTCTCTTACTGTCATGCCATCCGTAAGATGCTTTTCTGTGACTGGTGAGTACTCAACCAAGTCATTCTGAGAATAGTGTATGCGGCGACCGAGTTGCTCTTGCCCGGCGTCAATACGGGATAATACCGCGCCACATAGCAGAACTTTAAAAGTGCTCATCATTGGAAAACGTTCTTCGGGGCGAAAACTCTCAAGGATCTTACCGCTGTTGAGATCCAGTTCGATGTAACCCACTCGTGCACCCAACTGATCTTCAGCATCTTTTACTTTCACCAGCGTTTCTGGGTGAGCAAAAACAGGAAGGCAAAATGCCGCAAAAAAGGGAATAAGGGCGACACGGAAATGTTGAATACTCATACTCTTCCTTTTTCAATATTATTGAAGCATTTATCAGGGTTATTGTCTCATGAGCGGATACATATTTGAATGTATTTAGAAAAATAAACAAATAGGGGTTCCGCGCACATTTCCCCGAAAAGTGCCAC

##### vVA21 sequence

CTGACGCGCCCTGTAGCGGCGCATTAAGCGCGGCGGGTGTGGTGGTTACGCGCAGCGTGACCGCTACACTTGCCAGCGCCCTAGCGCCCGCTCCTTTCGCTTTCTTCCCTTCCTTTCTCGCCACGTTCGCCGTCCTTCAATGAAACATCGTTGGCCACTAATTTGGCCAGTGCAAAGTAGAACAAATCGGCAGCCTCCCAAGAAAGCTCCTTCTTACCCTTTGCCTCAGTCAGTTCTTCAGCTTCTTCCTTGATCTTGGCATCTAACAATGCAGAGTCGTTGAATAGTCTTCTAGTATAAGATTCCTCTGGAGCGTCCTGTAGCCTTTGTTTTAGTAAAGATTCTAGCCCCACCAAACCATGCTTGAATTCACCAAAGCAAGACATGGTCTCCAAGTGGCAAAATCCAACGTTTTCTTGTTCAACGATAAACTTTAAGGCATCCGAATCACAGTCAGTAGAGATTTGTAAAAGCTTTTGGCCATTGCCAGAAGTTTCACCCTTGATCCAGATTTCATTCCTAGAACGAGAATAATAAACGCCACGACCCAATTCGATGGCCTTTGCTATAGATTTCTTCGAAGAATACACCAACCCTAGACAACGCTCATATTGGTCCACAACTAGGGTGGTATATAAACCGTCAGGACGGTCTGTACGTACTTCACCAAGCACTTCTTTGGTCAACATATCCTTGCTTAATTTCTTTATGGACACAATTTTATCTTGCGAGAATTTTTGTTTTACCATGAATTGATTGGAGAAAACACCGTTCTCTTCCACAACAACACGCTCCTTTGGTACATTCAATTGTTCAACCAAGTGTTCGGCTGTTTTAGCATCTTGGCTTGCAATGAACAGAGAAGAAACTCCGTTGTTCAAGAAGGCAATGATTTCATCATCGCTGAATTTACCACTTGGCAAGGACAAAGCCACCAATGGAACTTCTTCCTCTTTGGAGAACTGGAGAATCTCTTCATTACTCAGGCTCGAGCCATCCAAAAGTACCTGACCAACAAGTGAAACGTATTCCTTCTTACTATTCCATGAGGCCAGATCATCAATTAACGGTAGAATCGGCAAAACCATTATTCAGAAAAAAAATTTTGTAAACTATTGTATTACTATTACACAGCGCAGTTGTGCTATGATATTAAAATGTATCCAGAACACACATCGGAGGTGAATATAACGTTCCATATCTATTATATACACAGTATACTACTGTTCATAGTCATATCCTTTTCTTACCTTCTATATCGAATGACTGATAATGCAACGTGAGTCACTGTGCATGGGTTTAGCAATTATTAAACTAATTTACCGGAGTCACTATTAGAGTCAGTTCGACTGCCTAGAAGAACTGCTGGTTGTCAGGATTGTGATGGGGGCATTCTGCTGTATTATGACCCATCGTATCGCAATGCTCACACCACTGTTGTCTTCCTGCCGTGGTATCGACTGGTGCAGGGGGGTCGAAAATTGATATACGGTACTACTGCACAACAAACTTTGGAAACCAACTTCAATGATCATCATGACTGCAATAAAAGCACTGAGAAACACGAGTTGATAATACCCACCCCATCAAAACCACTAAAGAAAAGGATATAAAGAAGACAAAGTAAAATGTATCAGCATTTACAACATTTGTCACGTTCTAAACCATTGCCGCTTACTCCAAACTCCAAATATAATGGGGAGGCTTGCGTCCAATTAGGGAAGACATATACAGTTATTCAGGATTACGAGCCTAGATTGACAGACGAAATAAGAATCTCGCTGGGTGAAAAAGTTAAAATTCTGGCCACTCATACCGATGGATGGTGTCTGGTAGAAAAGTGTAATACACAAAAGGGTTCTATTCACGTCAGTGTTGACGATAAAAGATACCTCAATGAAGATAGAGGCATTGTGCCTGGTGACTGTCTCCAAGAATACGACTGATGAAAATAATATTGACGTTCGCATTTAATCTATACCTATAATTCTGTACTTATATACTGTTCCTTAATTGAAGATTTCAACATCGTTTTTGATGTAGGTCTTTTCACCTGGAGGTGCGGCTGGGCTACCGAAGACTAATTGAGCTTGTACGGTCCAAGACTCAGGGATTTTGCTTGGCAAAGCAGCTTTTATGTAACCATTGTAGTGTTGTAGGTGACCACCCAGGCCCATTGCCTCCAAGGCAACCCACGAGTTGATTTGAGCGGCACCAGAGGTATGGTCCGCGAAACTAGGGAATGCAGCTGCGTACGCTGGGAAGTCAGCCTTTAGCTTTTCAGTTACCTTGTCGTCGGTGAAGAAGATTACAGAACCAAAGGCCTCATCCCTTGCTGAAGCAGGCCTCTTTTGACCGGCAGGGCTTTCTATAGCCTTAGTCACTTCGTCCCAAACTTTTTTGTGAGTTTCACCAGTCAAGATAACAGCGCGATTTGGCTGGGAGTTGAAAGCGGTGGGTGTGGCTCGAATGATGGTTTGGACGACGGATTGGATGTCGTTGATAGTAATTTCACCAGGTAACTCCGGTTTCAAAGCGTAAATAGTACGACGAGCAGTTAAAGTTTTCAAATAAGTTGCAACAGCAGACATGATATTGGATTGCCGGAATGGCGATATGTTGATCCCGGATACTTCAGTCTACGAAAAAAGTACAAATTATGTAGTCAGTTCCTTCAGTATGGTGTCCTTATATACTGTAGTTTGGACAAGGTGCAAATGCCAAGACCCTAGCCCGAAAAGCTCGAGGCACCCCAGGATCTTCCCCTTTACGTAATTTTCACGTAAAACGCCACAGTCCGATTTTTCTCGAATAATCATTAGTAAAAGCGGTATACTGGATTATTGTACGATAACAAGGTAGAGCTTTATTACTAAGCTAAGACGTTCTTACATCAATAGTGCTGTTCGTTATTGATGTTAGGAGAAGGAGCGGGTCTGGTGAATAGTGTAAGCAGTGTTTCTGAACTTTTTCTTCGTCTAAGTCCTTGTAATGTAAGGTAAGAATGCAAGCATCTTGTTTGTAACCCGGGTGTACGTTGACGTTAGTAAGTCACAAACCCAAGCTTAACTTCTTCGTGAGGAAGGAAAGTGTTGTGCTGAGGACTTAAGTGCTGAGGGAATTGTATTTATATTTATTTAGTACTTCTTGAGTTTACATATCCTTCGTAAAAATGCAACTTTTGTCGAAAAACACTTCCAAAAAAAAATAATAATGAATTTATGAAGCATACTAACGAGCGAGCACATCGCTGACCTATCATTACTTCATGAGATAAATTAAGATCTCCTCATATGCGAATTTCCTGTTCAGTGATAAACGTTGATTACGTTATTGATAAAAGTCTTTTCTTCTGGCAAGGGGTACCTGGAACACCAAAGACCAATTGAGATTGTACAGTCCACGCAATAGGAACATCTTGAGGCAAAGCAGATTTGACGTAGTCATTATAGTGTTGCAAATTAGCCCCCAATCCCAATAGTTCGAGGGCAGTCCAAGACTGAATTTGCACAGCACCGGTCGTATGAGCTCGCATGTTGGGAAAGCGGCTGCCAAGGCTGGAAATCTCTTTGCAGTTTTTCAGTTGGTCCTTCATCAGTGAGAAAATGACTGAACCGTAAGCCTCATCTCTGCAAGACTCTGGTCTCTTATAAAGCAGTTGGCATTGCGCTCGCAACAGCATCCCATATCCTTTTGTGTGTATCACCAACGATAATGACAGCGCGATTCACTTGTGAGTTAAAAGCTGTTGGCGTATTCTTGAGAATAACGTGTACAGTTCTCTTTACATCATCCAAACCGACACCTTGTGGTAATTCGGGCTTCAAATTGTAGATGGTACGACGGTTTGTAATAGCGTTTAAGTAGTTTCCAGTTGGGGACATTTCTTTGGCTTGGAGGTCTGGTGTTCTTGATTTTGATGGTGTATATAGCTTTAAAAAACCAAAAATGATCAACCTTTATATCGCTCTTCCGCTTCCTCGCTCACTGACTCGCTGCGCTCGGTCGTTCGGCTGCGGCGAGCGGTATCAGCTCACTCAAAGGCGGTAATACGGTTATCCACAGAATCAGGGGATAACGCAGGAAAGAACATGTGAGCAAAAGGCCAGCAAAAGGCCAGGAACCGTAAAAAGGCCGCGTTGCTGGCGTTTTTCCATAGGCTCCGCCCCCCTGACGAGCATCACAAAAATCGACGCTCAAGTCAGAGGTGGCGAAACCCGACAGGACTATAAAGATACCAGGCGTTTCCCCCTGGAAGCTCCCTCGTGCGCTCTCCTGTTCCGACCCTGCCGCTTACCGGATACCTGTCCGCCTTTCTCCCTTCGGGAAGCGTGGCGCTTTCTCATAGCTCACGCTGTAGGTATCTCAGTTCGGTGTAGGTCGTTCGCTCCAAGCTGGGCTGTGTGCACGAACCCCCCGTTCAGCCCGACCGCTGCGCCTTATCCGGTAACTATCGTCTTGAGTCCAACCCGGTAAGACACGACTTATCGCCACTGGCAGCAGCCACTGGTAACAGGATTAGCAGAGCGAGGTATGTAGGCGGTGCTACAGAGTTCTTGAAGTGGTGGCCTAACTACGGCTACACTAGAAGGACAGTATTTGGTATCTGCGCTCTGCTGAAGCCAGTTACCTTCGGAAAAAGAGTTGGTAGCTCTTGATCCGGCAAACAAACCACCGCTGGTAGCGGTGGTTTTTTTGTTTGCAAGCAGCAGATTACGCGCAGAAAAAAAGGATCTCAAGAAGATCCTTTGATCTTTTCTACGGGGTCTGACGCTCAGTGGAACGAAAACTCACGTTAAGGGATTTTGGTCATGAGATTATCAAAAAGGATCTTCACCTAGATCCTTTTAAATTAAAAATGAAGTTTTAAATCAATCTAAAGTATATATGAGTAAACTTGGTCTGACAGTTACCAATGCTTAATCAGTGAGGCACCTATCTCAGCGATCTGTCTATTTCGTTCATCCATAGTTGCCTGACTCCCCGTCGTGTAGATAACTACGATACGGGAGGGCTTACCATCTGGCCCCAGTGCTGCAATGATACCGCGAGACCCACGCTCACCGGCTCCAGATTTATCAGCAATAAACCAGCCAGCCGGAAGGGCCGAGCGCAGAAGTGGTCCTGCAACTTTATCCGCCTCCATCCAGTCTATTAATTGTTGCCGGGAAGCTAGAGTAAGTAGTTCGCCAGTTAATAGTTTGCGCAACGTTGTTGCCATTGCTACAGGCATCGTGGTGTCACGCTCGTCGTTTGGTATGGCTTCATTCAGCTCCGGTTCCCAACGATCAAGGCGAGTTACATGATCCCCCATGTTGTGCAAAAAAGCGGTTAGCTCCTTCGGTCCTCCGATCGTTGTCAGAAGTAAGTTGGCCGCAGTGTTATCACTCATGGTTATGGCAGCACTGCATAATTCTCTTACTGTCATGCCATCCGTAAGATGCTTTTCTGTGACTGGTGAGTACTCAACCAAGTCATTCTGAGAATAGTGTATGCGGCGACCGAGTTGCTCTTGCCCGGCGTCAATACGGGATAATACCGCGCCACATAGCAGAACTTTAAAAGTGCTCATCATTGGAAAACGTTCTTCGGGGCGAAAACTCTCAAGGATCTTACCGCTGTTGAGATCCAGTTCGATGTAACCCACTCGTGCACCCAACTGATCTTCAGCATCTTTTACTTTCACCAGCGTTTCTGGGTGAGCAAAAACAGGAAGGCAAAATGCCGCAAAAAAGGGAATAAGGGCGACACGGAAATGTTGAATACTCATACTCTTCCTTTTTCAATATTATTGAAGCATTTATCAGGGTTATTGTCTCATGAGCGGATACATATTTGAATGTATTTAGAAAAATAAACAAATAGGGGTTCCGCGCACATTTCCCCGAAAAGTGCCAC

##### vVA22 sequence

CTGACGCGCCCTGTAGCGGCGCATTAAGCGCGGCGGGTGTGGTGGTTACGCGCAGCGTGACCGCTACACTTGCCAGCGCCCTAGCGCCCGCTCCTTTCGCTTTCTTCCCTTCCTTTCTCGCCACGTTCGCCGTCCTTCAATGAAACATCGTTGGCCACTAATTTGGCCAGTGCAAAGTAGAACAAATCGGCAGCCTCCCAAGAAAGCTCCTTCTTACCCTTTGCCTCAGTCAGTTCTTCAGCTTCTTCCTTGATCTTGGCATCTAACAATGCAGAGTCGTTGAATAGTCTTCTAGTATAAGATTCCTCTGGAGCGTCCTGTAGCCTTTGTTTTAGTAAAGATTCTAGCCCCACCAAACCATGCTTGAATTCACCAAAGCAAGACATGGTCTCCAAGTGGCAAAATCCAACGTTTTCTTGTTCAACGATAAACTTTAAGGCATCCGAATCACAGTCAGTAGAGATTTGTAAAAGCTTTTGGCCATTGCCAGAAGTTTCACCCTTGATCCAGATTTCATTCCTAGAACGAGAATAATAAACGCCACGACCCAATTCGATGGCCTTTGCTATAGATTTCTTCGAAGAATACACCAACCCTAGACAACGCTCATATTGGTCCACAACTAGGGTGGTATATAAACCGTCAGGACGGTCTGTACGTACTTCACCAAGCACTTCTTTGGTCAACATATCCTTGCTTAATTTCTTTATGGACACAATTTTATCTTGCGAGAATTTTTGTTTTACCATGAATTGATTGGAGAAAACACCGTTCTCTTCCACAACAACACGCTCCTTTGGTACATTCAATTGTTCAACCAAGTGTTCGGCTGTTTTAGCATCTTGGCTTGCAATGAACAGAGAAGAAACTCCGTTGTTCAAGAAGGCAATGATTTCATCATCGCTGAATTTACCACTTGGCAAGGACAAAGCCACCAATGGAACTTCTTCCTCTTTGGAGAACTGGAGAATCTCTTCATTACTCAGGCTCGAGCCATCCAAAAGTACCTGACCAACAAGTGAAACGTATTCCTTCTTACTATTCCATGAGGCCAGATCATCAATTAACGGTAGAATCGGCAAAACCATTATTCAGAAAAAAAATTTTGTAAACTATTGTATTACTATTACACAGCGCAGTTGTGCTATGATATTAAAATGTATCCAGAACACACATCGGAGGTGAATATAACGTTCCATATCTATTATATACACAGTATACTACTGTTCATAGTCATATCCTTTTCTTACCTTCTATATCGAATGACTGATAATGCAACGTGAGTCACTGTGCATGGGTTTAGCAATTATTAAACTAATTTACCGGAGTCACTATTAGAGTCAGTTCGACTGCCTAGAAGAACTGCTGGTTGTCAGGATTGTGATGGGGGCATTCTGCTGTATTATGACCCATCGTATCGCAATGCTCACACCACTGTTGTCTTCCTGCCGTGGTATCGACTGGTGCAGGGGGGTCGAAAATTGATATACGGTACTACTGCACAACAAACTTTGGAAACCAACTTCAATGATCATCATGACTGCAATAAAAGCACTGAGAAACACGAGTTGATAATACCCACCCCATCAAAACCACTAAAGAAAAGGATATAAAGAAGACAAAGTAAAATGTATCAGCATTTACAACATTTGTCACGTTCTAAACCATTGCCGCTTACTCCAAACTCCAAATATAATGGGGAGGCTTGCGTCCAATTAGGGAAGACATATACAGTTATTCAGGATTACGAGCCTAGATTGACAGACGAAATAAGAATCTCGCTGGGTGAAAAAGTTAAAATTCTGGCCACTCATACCGATGGATGGTGTCTGGTAGAAAAGTGTAATACACAAAAGGGTTCTATTCACGTCAGTGTTGACGATAAAAGATACCTCAATGAAGATAGAGGCATTGTGCCTGGTGACTGTCTCCAAGAATACGACTGATGAAAATAATATTGACGTTCGCATTTAATCTATACCTATAATTCTGTACTTATATACTGTTCCTTAATTGAAGATTTCAACATCGTTTTTGATGTAGGTCTTTTCACCTGGAGGTGCGGCTGGGCTACCGAAGACTAATTGAGCTTGTACGGTCCAAGACTCAGGGATTTTGCTTGGCAAAGCAGCTTTTATGTAACCATTGTAGTGTTGTAGGTGACCACCCAGGCCCATTGCCTCCAAGGCAACCCACGAGTTGATTTGAGCGGCACCAGAGGTATGGTCCGCGAAACTAGGGAATGCAGCTGCGTACGCTGGGAAGTCAGCCTTTAGCTTTTCAGTTACCTTGTCGTCGGTGAAGAAGATTACAGAACCAAAGGCCTCATCCCTTGCTGAAGCAGGCCTCTTTTGACCGGCAGGGCTTTCTATAGCCTTAGTCACTTCGTCCCAAACTTTTTTGTGAGTTTCACCAGTCAAGATAACAGCGCGATTTGGCTGGGAGTTGAAAGCGGTGGGTGTGGCTCGAATGATGGTTTGGACGACGGATTGGATGTCGTTGATAGTAATTTCACCAGGTGCGGCCGCTTTCAAAGCGTAAATAGTACGACGAGCAGTTAAAGTTTTCAAATAAGTTGCAACAGCAGACATGATATTGGATTGCCGGAATGGCGATATGTTGATCCCGGATACTTCAGTCTACGAAAAAAGTACAAATTATGTGTCAGTTCCTTCAGTATGGTGTCCTTATATACTGTAGTTTGGACAAGGTGCAAATGCCAAGACCCTAGCCCGAAAAGCTCGAGGCACCCCAGGATCTTCCCCTTTACGTAATTTTCACGTAAAACGCCACAGTCCGATTTTTCTAGAATAATCATTAGTAAAAGCGGTATACTGGATTATTGTACGATAACAAGGTAGAGCTTTATTACTAAGCTAAGACGTTCTTACATCAATAGTGCTGTTCGTTATTGACGTCAGGAGAAGGAGCGGGTCTGGTGAATAGTGTAAGCAGTGTTTCTGAACTTTTTCTTCGTCTAAGTCCTTGTAATGTAAGGTAAGAATGCAAGCATCTTGTTTGTAACCCGGGTGTACGTTGACGTTAGTAAGGGGTGTACGTTGACGTTAGTAAGTCACAAACCCAAGCTTAACTTCTTCGTGAGGAAGGAAAGTGTTGTCTCCTACTTTTTTCAAATTTTCGAATTGTATTTATATTTATTTAGTACTTCTTGAGTTTACATATCCTTCGTAAAAATGCAACTTTTGTCGAAAAACACTTCCAAAAAAAAATAATAATGAATTTATGAAGCATACTAACGAGCGAGCACATCGCTGACCTATCATTACTTCATGAGATAAATTAAGATCTCCTCATATGCGAATTTCCTGTTCAGTGATAAACGTTGATTACGTTATTGATAAAAGTCTTTTCTTCTGGCAAGGGGTACCTGGAACACCAAAGACCAATTGAGATTGTACAGTCCACGCAATAGGAACATCTTGAGGCAAAGCAGATTTGACGTAGTCATTATAGTGTTGCAAATTAGCCCCCAATCCCAATAGTTCGAGGGCAGTCCAAGACTGAATTTGCACAGCACCGGTCGTATGAGCTCGCATGTTGGGAAAGCGGCTGCCAAGGCTGGAAATCTCTTTGCAGTTTTTCAGTTGGTCCTTCATCAGTGAAGAAAATGACTGAACCGTAAGCCTCATCTCTGCAAGACTCTGGTCTCTTATAAAGCAGTTGGCATTGCGCTCGCAACAGCATCCCATATCCTTTTGTGTGTATCACCAACGATAATGACAGCGCGATTCACTTGTGAGTTAAAAGCTGTTGGCGTATTCTTGAGAATAACGTGTACAGTTCTCTTTACATCATCCAAACCGACACCTTGTGGTAATTCGGGCTTCAAATTGTAGATGGTACGACGGTTTGTAATAGCGTTTAAGTAGTTTCCAGTTGGGGACATTTCTTTGGCTTGGAGGTCTGGTGTTCTTGATTTTGATGGTGTATATAGCTTTAAAAAACCAAAAATGATCAACCTTTATATCGCTCTTCCGCTTCCTCGCTCACTGACTCGCTGCGCTCGGTCGTTCGGCTGCGGCGAGCGGTATCAGCTCACTCAAAGGCGGTAATACGGTTATCCACAGAATCAGGGGATAACGCAGGAAAGAACATGTGAGCAAAAGGCCAGCAAAAGGCCAGGAACCGTAAAAAGGCCGCGTTGCTGGCGTTTTTCCATAGGCTCCGCCCCCCTGACGAGCATCACAAAAATCGACGCTCAAGTCAGAGGTGGCGAAACCCGACAGGACTATAAAGATACCAGGCGTTTCCCCCTGGAAGCTCCCTCGTGCGCTCTCCTGTTCCGACCCTGCCGCTTACCGGATACCTGTCCGCCTTTCTCCCTTCGGGAAGCGTGGCGCTTTCTCATAGCTCACGCTGTAGGTATCTCAGTTCGGTGTAGGTCGTTCGCTCCAAGCTGGGCTGTGTGCACGAACCCCCCGTTCAGCCCGACCGCTGCGCCTTATCCGGTAACTATCGTCTTGAGTCCAACCCGGTAAGACACGACTTATCGCCACTGGCAGCAGCCACTGGTAACAGGATTAGCAGAGCGAGGTATGTAGGCGGTGCTACAGAGTTCTTGAAGTGGTGGCCTAACTACGGCTACACTAGAAGGACAGTATTTGGTATCTGCGCTCTGCTGAAGCCAGTTACCTTCGGAAAAAGAGTTGGTAGCTCTTGATCCGGCAAACAAACCACCGCTGGTAGCGGTGGTTTTTTTGTTTGCAAGCAGCAGATTACGCGCAGAAAAAAAGGATCTCAAGAAGATCCTTTGATCTTTTCTACGGGGTCTGACGCTCAGTGGAACGAAAACTCACGTTAAGGGATTTTGGTCATGAGATTATCAAAAAGGATCTTCACCTAGATCCTTTTAAATTAAAAATGAAGTTTTAAATCAATCTAAAGTATATATGAGTAAACTTGGTCTGACAGTTACCAATGCTTAATCAGTGAGGCACCTATCTCAGCGATCTGTCTATTTCGTTCATCCATAGTTGCCTGACTCCCCGTCGTGTAGATAACTACGATACGGGAGGGCTTACCATCTGGCCCCAGTGCTGCAATGATACCGCGAGACCCACGCTCACCGGCTCCAGATTTATCAGCAATAAACCAGCCAGCCGGAAGGGCCGAGCGCAGAAGTGGTCCTGCAACTTTATCCGCCTCCATCCAGTCTATTAATTGTTGCCGGGAAGCTAGAGTAAGTAGTTCGCCAGTTAATAGTTTGCGCAACGTTGTTGCCATTGCTACAGGCATCGTGGTGTCACGCTCGTCGTTTGGTATGGCTTCATTCAGCTCCGGTTCCCAACGATCAAGGCGAGTTACATGATCCCCCATGTTGTGCAAAAAAGCGGTTAGCTCCTTCGGTCCTCCGATCGTTGTCAGAAGTAAGTTGGCCGCAGTGTTATCACTCATGGTTATGGCAGCACTGCATAATTCTCTTACTGTCATGCCATCCGTAAGATGCTTTTCTGTGACTGGTGAGTACTCAACCAAGTCATTCTGAGAATAGTGTATGCGGCGACCGAGTTGCTCTTGCCCGGCGTCAATACGGGATAATACCGCGCCACATAGCAGAACTTTAAAAGTGCTCATCATTGGAAAACGTTCTTCGGGGCGAAAACTCTCAAGGATCTTACCGCTGTTGAGATCCAGTTCGATGTAACCCACTCGTGCACCCAACTGATCTTCAGCATCTTTTACTTTCACCAGCGTTTCTGGGTGAGCAAAAACAGGAAGGCAAAATGCCGCAAAAAAGGGAATAAGGGCGACACGGAAATGTTGAATACTCATACTCTTCCTTTTTCAATATTATTGAAGCATTTATCAGGGTTATTGTCTCATGAGCGGATACATATTTGAATGTATTTAGAAAAATAAACAAATAGGGGTTCCGCGCACATTTCCCCGAAAAGTGCCAC

#### Pol ε and Pol δ mutants yeast expression strain construction

The endogenous *pol2* gene was modified in yJF1 (Frigola et al., 2013) strain to add a C-terminal 3xFLAG tag by transformation with PCR-amplified product, using the oligonucleotides VA_oligo_1 and VA_oligo_2, and pBP83 (Yeeles et al., 2015) as template. Pol2 tagged protein (yVA2) was verified by immunoblot. yVA2 strain was transformed with the linearized expression vector pRS306 (Dpb2 + Dpb3), to integrate Pol ε subunits Dpb2 and Dpb3. Pol2 gene mutations F1199A, F1200A (Pol ε^PIP^) and D640A (Pol ε^Cat^) were obtained by site directed mutagenesis, and subsequently sub-cloned into pRS304 (Pol2 + Dpb4-CBP) (Yeeles et al., 2015). The Pol 2 F1199A, F1200A / D680A construct was obtained by sub-cloning the DNA fragment containing F1199A, F1200A mutation into Pol 2 D640A expression vector. Pol3 coding sequence in pRS306/Pol3- Gal-Pol31 (Yeeles et al., 2017) was mutated by site directed mutagenesis to generate Pol3 D608A mutation (vVA5). yJF1 was transformed sequentially with pRS303/Pol32- CBP-Gal-Gal4 and vVA5 to generate yVA28. Sequencing analysis (Source BioScience) verified the success of cloning and the absence of undesired mutations in the gene coding sequences. Details of oligonucleotides and plasmids are reported in Key Resource Table. More information about expression strains can be found in Table S1 and Key Resource Table.

#### Purification of replication proteins

With the exception of the Pol ε and Pol δ mutants (see details below), all proteins were purified as described previously (Taylor and Yeeles, 2018, Yeeles et al., 2015, Yeeles et al., 2017). A list of the proteins used in this work and a summary of the their purification steps are described in Key Resource Table and in Table S2, respectively.

**Pol ε mutants** (**Pol ε**^**PIP**^**, Pol ε**^**Cat**^**, Pol ε**^**PIP/Cat**^ purification) were expressed and purified in *S. cerevisiae* essentially as for wild-type Pol ε (Yeeles et al., 2015). To remove endogenous Pol2 from the preparations the eluate from the CBP column was incubated with anti-FLAG M2 Affinity Gel (Sigma) for 1 hour at 4°C. The flow through was collected and the purification continued as for Pol ε (Yeeles et al., 2015).

Pol δ^Cat^ was purified from *S. cerevisiae*, as reported for the wild-type protein (Yeeles et al., 2017), with some modifications. Lysed cells were diluted 4 times in Buffer T (25 mM Tris-HCl, pH 7.2; 10% glycerol; 0.02% NP-40-S) + 400 mM NaCl + 1 mM DTT, supplemented with cOmplete, EDTA-free protease inhibitor cocktail (Roche). Cell extract was clarified by ultracentrifugation (235,000 g, 50 min, 4°C) and 0.025% Polymin P was added to the soluble material, while stirring at 4°C for 10 minutes. After centrifugation (27,000 g, 4°C, 15 min) the soluble material was precipitated with ammonium sulfate (0.28 g/ml) and further centrifuged, as before. The resulting pellet was resuspended in Buffer T + 300 mM NaCl + 2 mM CaCl_2_ + 1 mM DTT + protease inhibitor cocktail, and incubated with Calmodulin-Sepharose 4B for 2 hours at 4°C. The resin was washed with 100 CV of Buffer T + 300 mM NaCl +2 mM CaCl_2_ + 1 mM DTT, and with a further 10 CV buffer without NP-40-S and with reduced NaCl (200 mM). Protein elution was carried out with 10 CV of Buffer T + 200 mM NaCl, supplemented with 2 mM EDTA and 2 mM EGTA. Eluted protein was diluted 1:2 in Buffer T lacking NaCl to reduce the salt concentration tot 100 mM, and then loaded on MonoQ column (GE Healthcare). The column was washed with Buffer T containing 100 mM NaCl and Pol δ^Cat^ was eluted with 20 CV of 100-600 mM NaCl. The peak fraction was dialysed against storage buffer (25 mM HEPES-KOH, pH 7.6; 40% glycerol; 300 mM KOAc; 0.02% NP-40-S; 1 mM DTT).

#### Primer extension assay

Primer extension reactions were essentially performed as described previously but using M13-mp18 ssDNA (NEB), rather than ϕX174 (Schauer and O’Donnell, 2017). Singularly primed M13-mp18 template was prepared as previously reported (Yeeles et al., 2017). Template (1.5 nM) was incubated with 600 nM RPA for 10 minutes at 30°C in a buffer containing 25 mM Tris-Acetate, pH 7.5; 5% glycerol; 5 mM DTT; 8 mM Mg(OAc)_2_; 50 mM potassium glutamate; 0.5 mM ATP; 60 μM dATP; 60 μM dGTP. Protein mix containing PCNA and Pol ε or Pol ε^PIP^ (20 nM final concentrations after mixing) was added to RPA-bound template. The two protein-DNA mixtures were split and increasing concentrations of RFC were added. The reactions were incubated at 30°C for 5 minutes and DNA synthesis started by adding nucleotide mix (60 μM of each dTTP and dCTP and 1 μCi [α-^32^P]-dCTP). After 12 minutes at 30°C, the reactions were stopped with stop solution (25 mM EDTA, 0.5% SDS) and separated on 0.7% alkaline agarose gels.

#### Standard replication reactions on naked template

Replication reactions were essentially conducted as previously described (Taylor and Yeeles, 2018). MCM loading and phosphorylation was performed at 24°C in a reaction (20-40 μl) containing: 25 mM HEPES-KOH, pH 7.6; 100 mM potassium glutamate; 0.01% NP-40-S; 1 mM DTT; 10 mM Mg(OAc)_2_; 0.1 mg/ml BSA; 3 mM ATP; 5 nM DNA template; 75 nM Cdt1-Mcm2-7; 40 nM Cdc6; 20 nM ORC; 25 nM DDK. After a 10 min incubation S-CDK was added to 100 nM and incubation prolonged for a further 5 min. The loading reaction was 4-fold diluted into replication buffer containing the following components, reported in their final concentrations: 25 mM HEPES-KOH, pH 7.6; 250 mM potassium glutamate; 0.01% NP-40-S; 1 mM DTT; 10 mM Mg(OAc)_2_; 0.1 mg/ml BSA; 3 mM ATP; 200 μM CTP; 200 μM GTP; 200 μM UTP; 30 μM dATP; 30 μM dCTP; 30 μM dGTP; 30 μM dTTP; 1 μCi [α-^32^P]-dCTP. Samples were pre-warmed at 30°C for 1 minute and reactions were initiated by addition of replication proteins to the following concentrations: 30 nM Dpb11; 100 nM GINS; 30 nM Cdc45; 10 nM Mcm10; 20 nM Ctf4; 20 nM Csm3/Tof1; 20 nM RFC; 20 nM PCNA; 20 nM Pol ε (or mutants); 10 nM Pol δ (or Pol δ^Cat^); 100 nM RPA; 20 nM Pol α; 10 nM Topoisomerase I; 10 nM Mrc1; 12.5 nM Sld3/7; 30 nM Sld2. The samples were incubated at 30°C for the indicated times and were processed and imaged as described (Yeeles et al., 2017) (see Gel processing for details).

#### Pulse-chase experiments on naked templates

Reactions were performed as for standard replication reactions except that the dCTP concentration during the pulse was lowered to 2.5 μM. For the chase the dCTP concentration was increased to 600 μM by addition of dCTP from a 50 mM stock.

#### Replication reactions on chromatinized templates

Templates were chromatinized essentially as described (Kurat et al., 2017) with minor modifications. Chromatin assembly was performed in a reaction (40 μl) containing 25 mM HEPES-KOH, pH 7.6; 100 mM KOAc; 5% glycerol; 10 mM Mg(OAc)_2_; 0.1 mg/ml BSA; 3 mM ATP; 40 mM Creatine Phosphate; 0.28 mg/ml Creatine Phosphate Kinase; 30 nM Isw1a; 3 μM Nap1; 370 nM Histones. The reaction was incubated on ice before DNA template was added to 3 nM. Following a 10 min incubation at 30°C ORC was added to 20 nM and incubation continued for 50 min. Chromatinized template was isolated by centrifugation (750 g, room temperature, 2 min) through ∼400 μL Sephacryl-S400 High Resolution (GE Healthcare) columns, previously equilibrated in 25 mM HEPES-KOH, pH 7.6; 100 mM potassium glutamate; 0.01% NP-40-S; 1 mM DTT; 10 mM Mg(OAc)_2_; 40 mM KCl. MCM loading and phosphorylation was performed by adding (final concentrations are given) 3 mM ATP; 0.1 mg/ml BSA; 75 nM Cdt1-Mcm2-7; 40 nM Cdc6; 25 nM DDK. After 10 minutes at 24°C, S-CDK was added to 25 mM and incubation continued for 5 minutes. The nucleotide components were added (final concentrations reported as in Standard replication reactions) and the mix was pre-warmed at 30°C for 1 minute. Reactions were initiated by addition of replication proteins as described for standard replication reactions. Nhp6 and FACT were also added to final concentrations of 400 nM and 40 nM respectively. For the experiments on the ARS1 template the DDK concentration was 50 nM, the Sld3/7 concentration was 25 nM, the GINS concentration was 200 nM, the Mcm10 concentration was 5 nM and the RPA concentration was 60 nM. After quenching by addition of an equal volume of 50 mM EDTA, proteins were removed from the replication mix by proteinase K (8 U/ml, NEB) – SDS (0.1%) treatment, for 15 minutes at 37°C, followed by phenol-chloroform-isoamyl alcohol extraction (Sigma-Aldrich). Unincorporated nucleotide was removed using Illustra G-50 columns (GE Healthcare). Samples were analyzed as described (Taylor and Yeeles, 2018) by native, denaturing and two-dimensional agarose gel electrophoresis, where required.

#### Leading-strand initiation site mapping

To determine the distribution of nascent leading-strand initiation sites, deproteinized replication products from chromatin reactions were cleaved with the restriction enzymes XbaI, NotI, SacI, PsiI, EcoRV or BsaBI (NEB) in Cut Smart buffer, for 30 minutes at 37°C. Digests were stopped by adding EDTA to final concentration of 50 mM, followed by deprotinization with proteinase K - SDS treatment and phenol-chloroform extraction as described above. Sample aliquots were analyzed on 1% alkaline and 0.8% native agarose gels, where required. The remaining digested products were ethanol precipitated, washed with 70% ethanol, air-dried and resuspended in 10 mM Tris-HCl, pH 8; 1 mM EDTA. For the RNase HII experiments, digestion products were further treated with RNase HII enzyme (NEB) for 1 hour at 37°C. The reactions were stopped with 50 mM EDTA and processed as described above for the restriction digests. For polyacrylamide gel analysis an equal volume of 2x loading dye (80% formamide; 0.05% SDS; 10 mM EDTA; 100 mM NaCl; 0.04% xylene cyanol; 0.04% bromophenol blue) was added to the samples. Samples were incubated for 3 minutes at 95°C, promptly transferred to ice, before being applied to a 40 cm x 20 cm denaturing 4% polyacrylamide (Bis-Acrylamide 19:1 – Fisher scientific), 7 M Urea, in 1x Tris-Borate-EDTA buffer (TBE) gel. Gels were run for 170 minutes at constant 40 Watt.

#### Gel processing

Native agarose gels and acrylamide gels were dried directly onto 3MM chromatography paper (GE Healthcare). Alkaline agarose gels were fixed with two 15 min incubations at 4°C in 5% trichloroacetic acid solution before drying on 3MM chromatography paper (GE Healthcare). For quantification, gels were exposed on BAS-IP MS Storage Phosphor Screens (GE Healthcare) and imaged on a Typhoon phophorimager (GE Healthcare). Gels were also autoradiographed using Amersham Hyperfilm MP (GE Healthcare) for presentation.

### Quantification and Statistical Analysis

Quantification and data analysis were performed with ImageJ software and Prism 7. For pulse-chase experiments to determine maximum leading-strand synthesis rates (Figures 1 and S1) lane profiles were first generated in ImageJ. To assign the maximum product length straight lines were manually fit to the lane background and the ‘front’ of the leading-strand population. The intercept between these lines was taken as the migration position for maximum length leading-strand products. Migration positions were converted to product lengths using a standard curve generated from the molecular weight markers. Data were fit to linear regressions and the slope of the regression was used to calculate replication rates (Yeeles et al., 2017).

For the initiation site mapping experiments lanes profiles were generated in ImageJ. The migration positions of the replication products were converted to product lengths using standard curves generated from the molecular weight markers. To generate the standard curve the migration position of the marker bands were plot against the Log10 of their length and data were fit to a second order polynomial. Signal from the no enzyme lanes was subtracted from the profiles and data were normalized by dividing by the maximum peak value for a given profile. Initiation site positions relative to the 5ʹ end of the ACS were then derived by subtracting the distance between the enzyme cleavage site and the 5ʹ end of the ACS.

## Supplemental Information

Document S1. Figures S1–S6 and Tables S1 and S2

Document S2. Article plus Supplemental Information
