# Supplementary material for: Mechanism of Bidirectional Leading-Strand Synthesis Establishment at Eukaryotic DNA Replication Origins
Source: Mol Cell. 2019 Jan 17;73(2):199–211.e10. doi: 10.1016/j.molcel.2018.10.019 (PMC6344338; doi:10.1016/j.molcel.2018.10.019)
Supplement: Document S1. Figures S1–S6 and Tables S1 and S2 [file mmc1.pdf]

Molecular Cell, Volume *73*

Supplemental Information

Mechanism of Bidirectional Leading-Strand Synthesis Establishment at Eukaryotic DNA Replication Origins

Valentina Aria and Joseph T.P. Yeeles

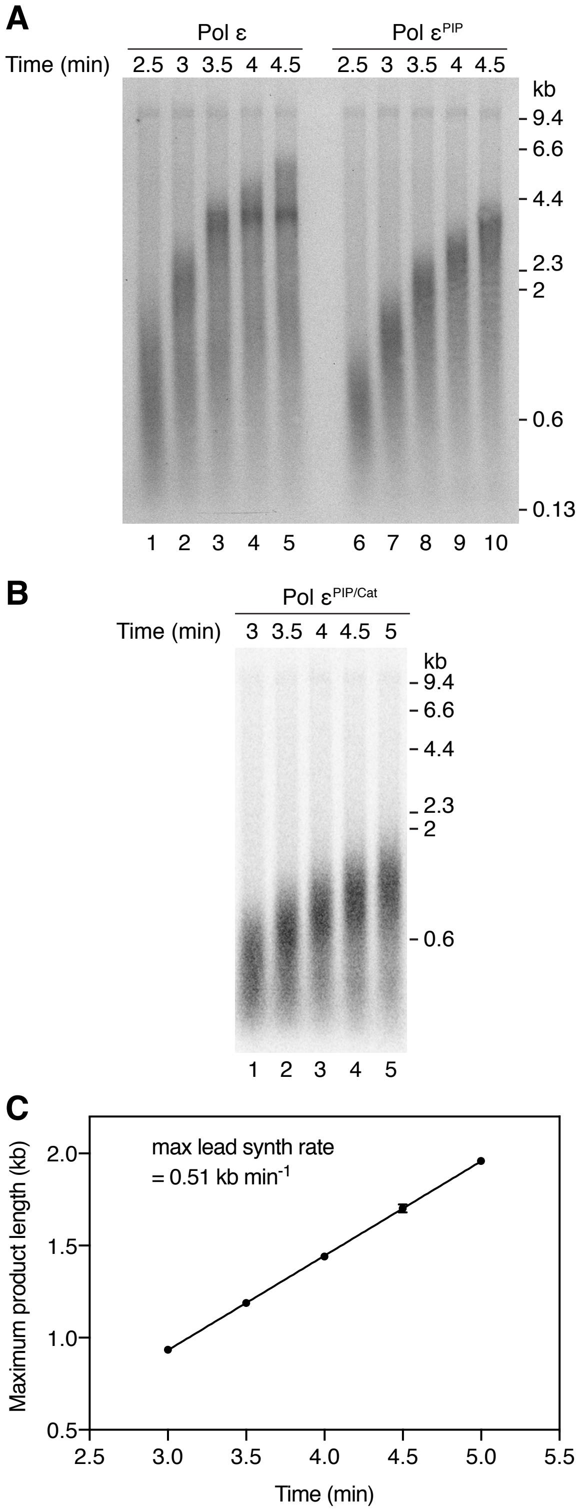

Figure S1. Related to Figure 1.

(A and B) Pulse-chase experiments performed on the template illustrated in Figure 1C in the presence of Pol δ. The chase was added at 2 min 20 s (A) and 2 min 50 s (B) respectively.

(C) Quantitation of pulse-chase experiments performed as in (B). Error bars represent the SEM from 2 experiments.

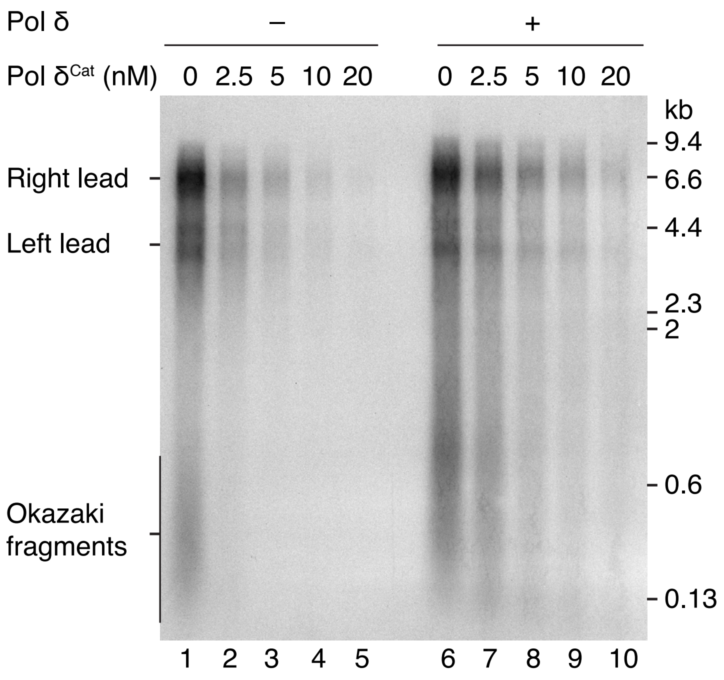

Figure S2. Related to Figure 2.

Pol δ^{Cat} titration in the presence and absence of Pol δ (10 nM). The reaction was performed on the template illustrated in Figure 1C with 20 nM Pol ε. Reactions were incubated for 12 min and products were separated through a 1% alkaline agarose gel.

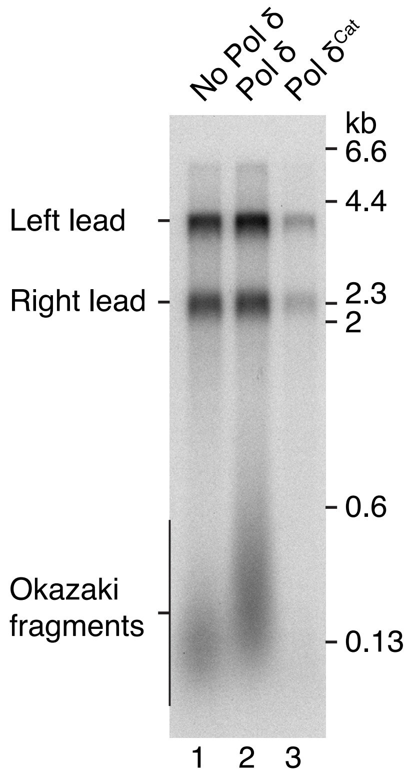

Figure S3. Related to Figure 3.

Chromatin replication reaction (60 min) performed on the template illustrated in Figure 3A.

Products were analysed through a 1% alkaline agarose gel.

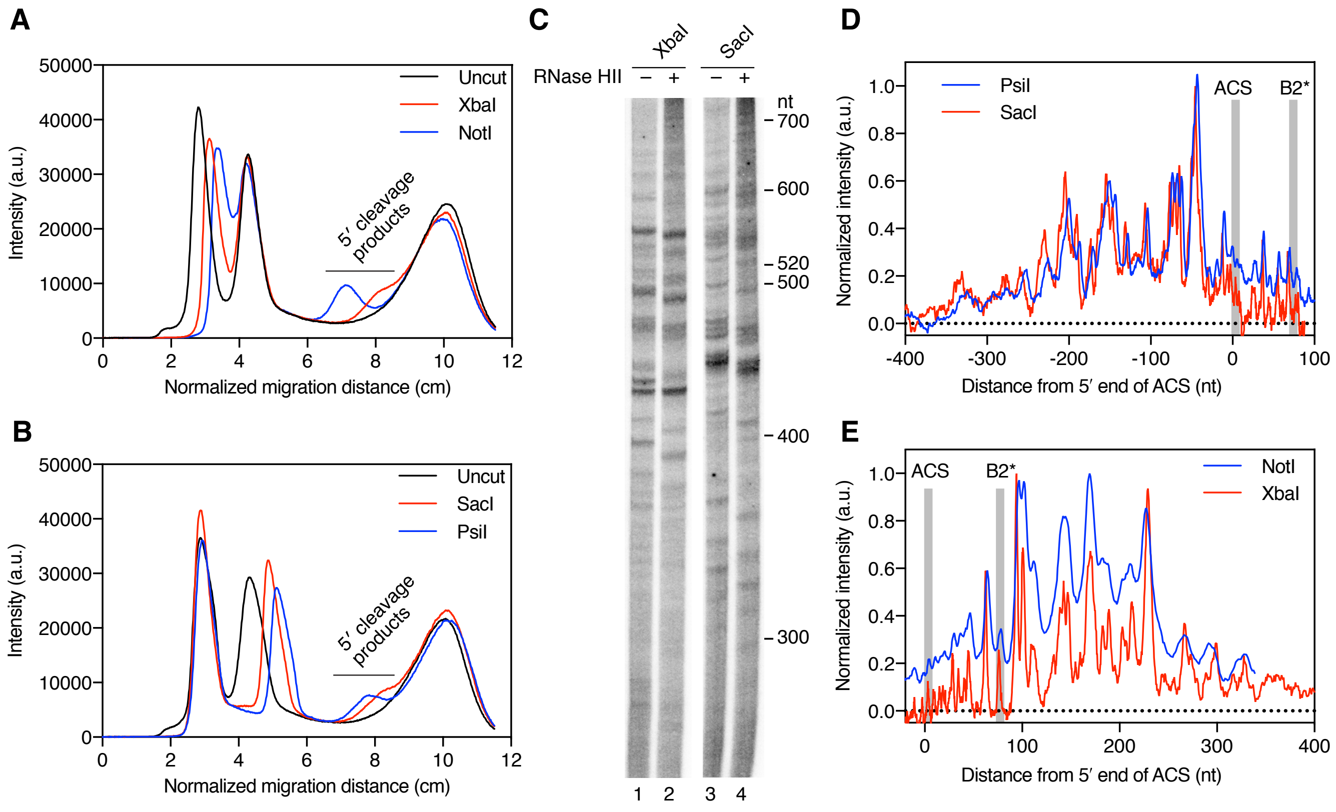

Figure S4. Related to Figure 3.

(A) Lane scans for the data in Figure 3B lanes 1-3.

(B) Lane scans for the data in Figure 3B lanes 4-6.

(C) XbaI (Left lead) and SacI (Right lead) digested products from the reaction in Figure 3B were digested with RNase HII and separated through a 4% denaturing polyacrylamide gel.

(D and E) Normalized lane scans of the data in Figure 3C for enzymes that digest the ‘Right’ (D) and ‘Left’ (E) leading strands.

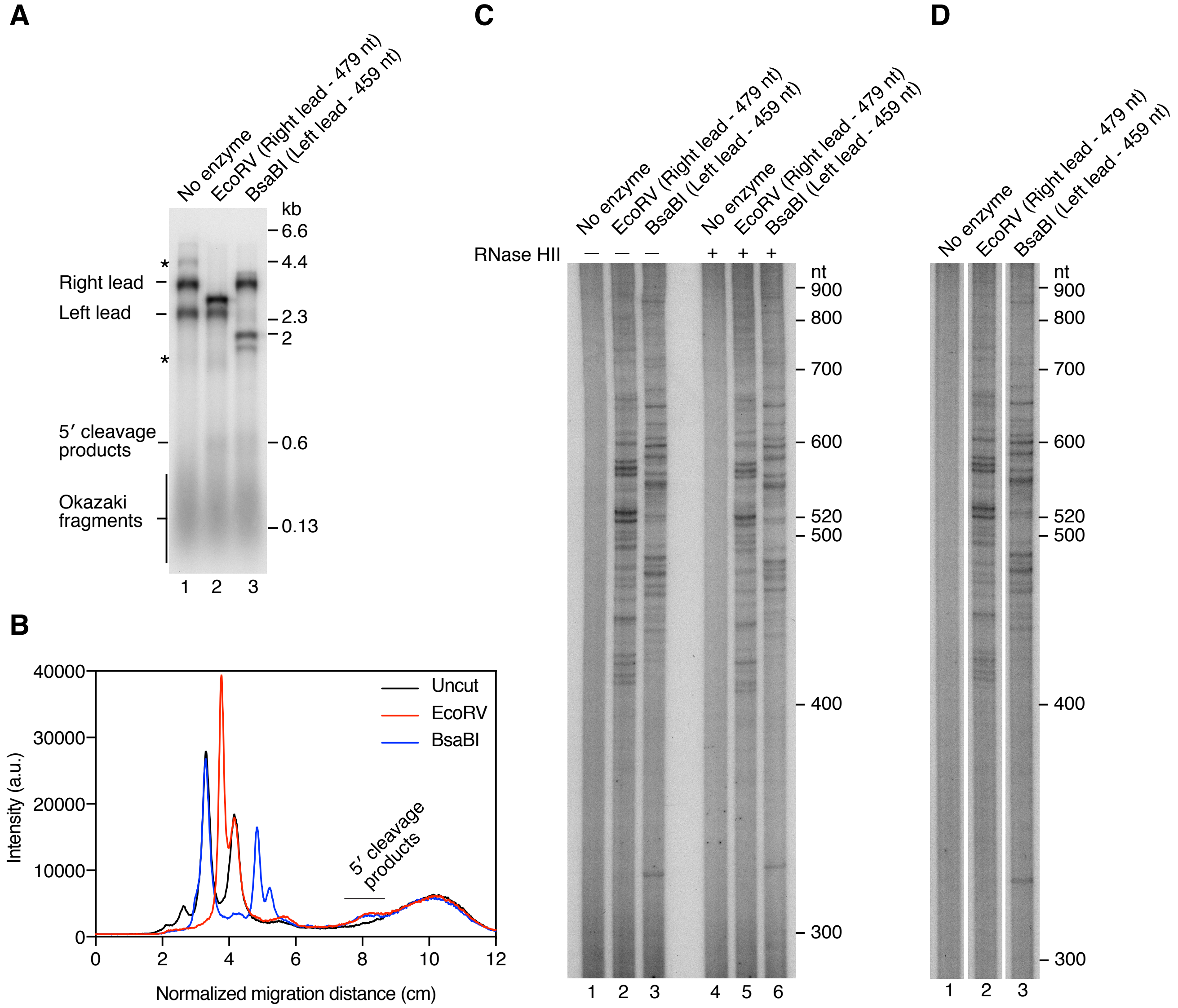

Figure S5. Related to Figure 4.

(A) Replication reaction performed on the chromatinized template in Figure 4A for 60 min. Products were separated through a 1% alkaline agarose gel. * marks the positions of replication products likely generated from activation of MCM loaded outside ARS1.

(B) Lane profiles of the data in (A).

(C) Digested replication products from (A) were either mock treated or treated with RNase HII prior to being separated through a 4% denaturing polyacrylamide gel.

(D) Replication reaction performed and analyzed as in (C) in the absence of RNase HII.

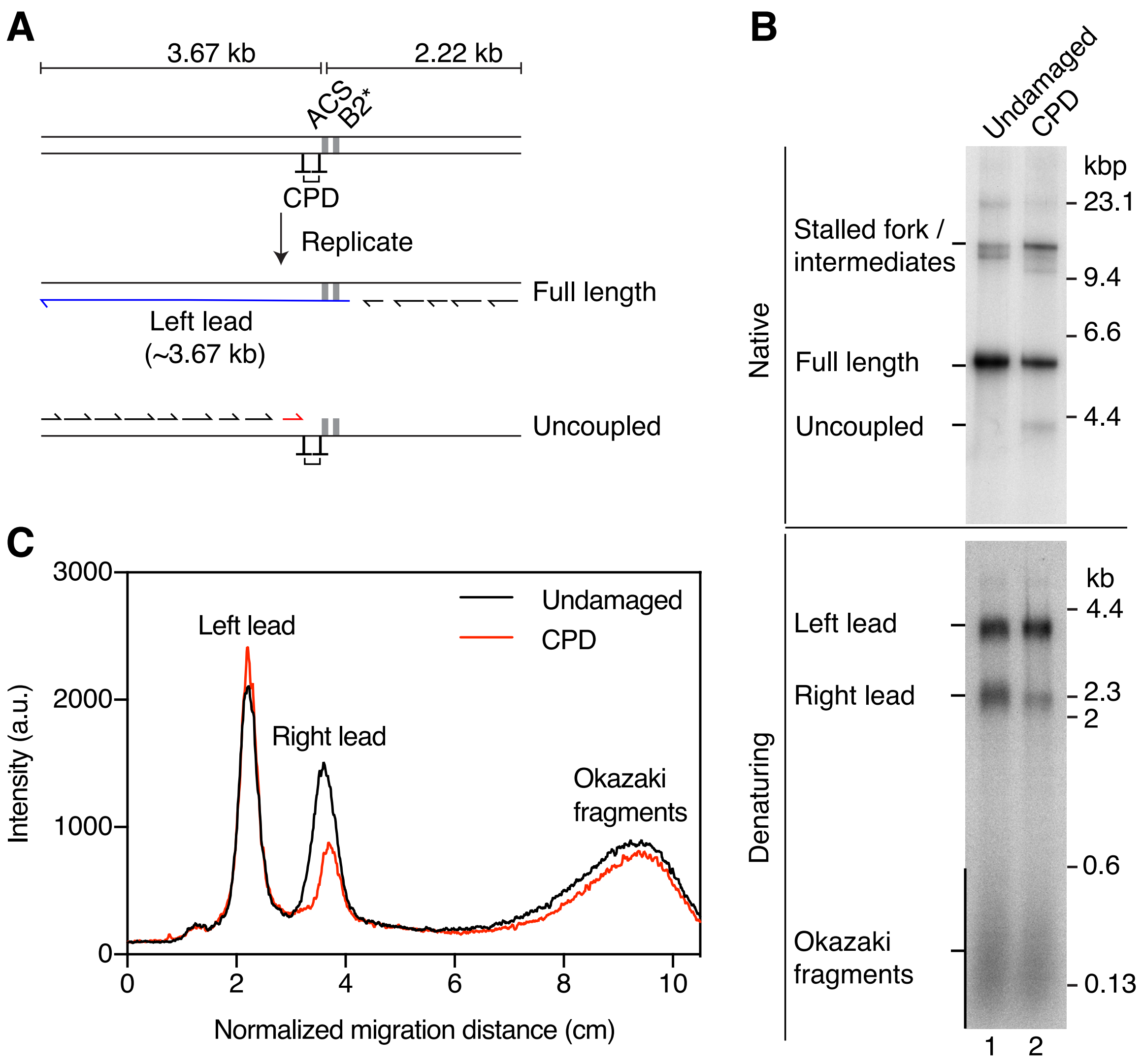

Figure S6. Related to Figure 6.

(A) Illustration of a CPD-containing ARS306 template.

(B) Chromatin replication reaction performed on the template illustrated in (A) and its undamaged equivalent. Products were analysed through 0.8% native and 1% denaturing agarose gels as indicated.

(C) Lane profiles of the data in (B), denaturing.

Strain	Genotype
yVA2	*MATa ade2-1 ura3-1 his3-11,15 trp1-1 leu2-3,112 can1-100*
	bar1::Hyg
	pep4::KanMX
	pol2 C-ter 3xFLAG Nat NT2
yVA7	*MATa ade2-1 ura3-1 his3-11,15 trp1-1 leu2-3,112 can1-100*
	bar1::Hyg
	pep4::KanMX
	pol2 C-ter 3xFLAG Nat NT2
	ura3::URA3pRS306/Dpb2, Dpb3
	trp1::TRP1pRS304/Pol2(D640A), Dpb4-Tev-CBP
yVA11	*MATa ade2-1 ura3-1 his3-11,15 trp1-1 leu2-3,112 can1-100*
	bar1::Hyg
	pep4::KanMX
	pol2 C-ter 3xFLAG Nat NT2
	ura3::URA3pRS306/Dpb2, Dpb3
	trp1::TRP1pRS304/Pol2(F1199A-F1200A), Dpb4-Tev-CBP
yVA26	*MATa ade2-1 ura3-1 his3-11,15 trp1-1 leu2-3,112 can1-100*
	bar1::Hyg
	pep4::KanMX
	pol2 C-ter 3xFLAG Nat NT2
	ura3::URA3pRS306/Dpb2, Dpb3
	trp1::TRP1pRS304/Pol2(D640A- F1199A-F1200A), Dpb4-Tev-CBP
yVA28	*MATa ade2-1 ura3-1 his3-11,15 trp1-1 leu2-3,112 can1-100*
	bar1::Hyg
	pep4::KanMX
	ura3::URA3pRS306/Pol31, Pol3 (D608A)
	his3::HIS3pRS303/Pol32-CBP

Table S1. Related to STAR Methods.

Yeast strains for protein expression.

Protein	Tag	Purification steps
Cdc45	Internal 2xFLAG tag	Anti-FLAG M2 Agarose<br>Bio-Gel HT Hydroxyapatite
Cdc6	N-terminal GST cleavable tag	Glutathione Sepharose 4B<br>Bio-Gel HT Hydroxyapatite
Cdt1-Mcm2-7	N-terminal CBP cleavable tag on Mcm3	Calmodulin-Sepharose 4B<br>Superdex 200
Csm3/Tof1	N-terminal CBP cleavable tag on Csm3	Calmodulin-Sepharose 4B<br>TEV removal with Talon column<br>Superdex 200
Ctf4	N-terminal CBP tag	Calmodulin-Sepharose 4B<br>MonoQ<br>Superdex 200
DDK	CBP tag on Dbf4	Calmodulin-Sepharose 4B<br>Lambda phosphatase dephosphorylation<br>Superdex 200
Dpb11	C-terminal 3xFLAG tag	Anti-FLAG M2 Agarose<br>MonoS
FACT	His tag	Talon column<br>MonoQ
GINS	N-terminal His tag on Psf3	Ni-NTA Agarose<br>MonoQ<br>Superdex 200
Histones	Untagged	HiTrap Heparin HP<br>Superdex 200
Isw1a	C-terminal 3xFLAG tag	Anti-FLAG M2 Agarose<br>MonoQ
Mcm10	N-terminal His tag	Ni-NTA Agarose<br>MonoS (twice)
Mrc1	C-terminal 2xFLAG tag	Anti-FLAG M2 Agarose<br>MonoQ
Nap1	GST cleavable tag	Glutathione Sepharose 4B<br>MonoQ
Nhp6	Untagged	Protein precipitation with TCA<br>HiTrap SP HP column
ORC	CBP-cleavable tag on Orc1	Calmodulin-Sepharose 4B<br>Superdex 200
PCNA	Untagged	Nucleic acid precipitation with Polymin P<br>Ammonium sulfate precipitation<br>HiTrap SP HP (flow through)<br>HiTrap Heparin HP (flow through)<br>HiTrap DEAE Fast Flow<br>MonoQ

Pol α	N-terminal CBP tag on Pri1	Calmodulin-Sepharose 4B<br>MonoQ<br>Superdex 200
Pol δ	C-terminal CBP tag on Pol32	Calmodulin-Sepharose 4B<br>HiTrap Heparin HP<br>Superdex 200
Pol ε	C-terminal CBP tag on Dpb4	Calmodulin-Sepharose 4B<br>HiTrap Heparin HP<br>Superdex 200
RFC	N-terminal CBP tag on Rfc3	Calmodulin-Sepharose 4B<br>MonoS<br>Superdex 200
RPA	Untagged	HiTrap Blue HP<br>ssDNA Cellulose<br>MonoQ
Sld2	C-terminal 3x FLAG	Ammonium sulfate precipitation<br>Anti-FLAG M2 Agarose<br>HiTrap SP HP
Sld3/7	C-terminal cleavable TCP tag	IgG Sepharose Fast Flow<br>TEV removal with Ni-NTA Agarose<br>Superdex 200
TopoI	N-terminal cleavable CBP tag	Calmodulin-Sepharose 4B<br>TEV removal with Talon column<br>Superdex 200

Table S2. Related to STAR Methods.

Purification strategy used for replication proteins.
